# Independence of chromatin conformation and gene regulation during *Drosophila* dorsoventral patterning

**DOI:** 10.1038/s41588-021-00799-x

**Published:** 2021-04-01

**Authors:** Elizabeth Ing-Simmons, Roshan Vaid, Xin Yang Bing, Michael Levine, Mattias Mannervik, Juan M. Vaquerizas

**Affiliations:** 1 Max Planck Institute for Molecular Biomedicine, Münster, Germany; 2 Department of Molecular Biosciences, The Wenner-Gren Institute, Stockholm University, Stockholm, Sweden; 3 Lewis-Sigler Institute for Integrative Genomics, Princeton University, Princeton, NJ USA; 4 Department of Molecular Biology, Princeton University, Princeton, NJ USA; 5 MRC London Institute of Medical Sciences, Institute of Clinical Sciences, Faculty of Medicine, Imperial College London, London, UK

**Keywords:** Gene regulation, Epigenomics, Cell lineage, Pattern formation, Epigenetics

## Abstract

The relationship between chromatin organization and gene regulation remains unclear. While disruption of chromatin domains and domain boundaries can lead to misexpression of developmental genes, acute depletion of regulators of genome organization has a relatively small effect on gene expression. It is therefore uncertain whether gene expression and chromatin state drive chromatin organization or whether changes in chromatin organization facilitate cell-type-specific activation of gene expression. Here, using the dorsoventral patterning of the *Drosophila melanogaster* embryo as a model system, we provide evidence for the independence of chromatin organization and dorsoventral gene expression. We define tissue-specific enhancers and link them to expression patterns using single-cell RNA-seq. Surprisingly, despite tissue-specific chromatin states and gene expression, chromatin organization is largely maintained across tissues. Our results indicate that tissue-specific chromatin conformation is not necessary for tissue-specific gene expression but rather acts as a scaffold facilitating gene expression when enhancers become active.

## Abstract (web summary)

Chromatin conformation is largely independent of dorsoventral gene expression during early embryonic development in *Drosophila*. Despite tissue-specific differences in chromatin state and gene expression, three-dimensional chromatin organization is maintained across tissues.

## Main

Chromatin is highly organized within discrete chromosome territories, into compartments of active or inactive chromatin, self-interacting domains, and loops between specific loci (reviewed in refs. ^1,2^). However, the relationship between the three-dimensional (3D) organization of chromatin and the regulation of gene expression remains unclear. There is considerable evidence that chromatin conformation is important for gene regulation: disruption of domains and domain boundaries can lead to misexpression of developmental genes, contributing to developmental defects or cancer^3–8^. In addition, the general principles of 3D genome organization are conserved across large evolutionary distances, as well as the chromatin conformation at specific loci^9–14^. Further evidence comes from the identification of interactions between promoters and their regulatory elements^15–22^ and the finding that forced enhancer–promoter looping is sufficient to activate transcription of some genes^23–26^. However, in other cases, direct enhancer–promoter contacts may be neither strictly required nor sufficient for gene activation^27–30^. Furthermore, depletion of key regulators of genome organization, such as CCCTC-binding factor (CTCF) or cohesin, has relatively small effects on gene expression^31–34^, and genomic rearrangements are not always associated with changes in gene expression^35–38^.

While multiple studies documented differences in chromatin conformation between different cell types or tissues^39–45^, it is not known whether these changes are the cause or consequence of changes in gene expression. Therefore, a fundamental question arises as to whether changes in gene expression and chromatin state drive chromatin reorganization, or whether changes in chromatin organization facilitate cell-type-specific activation of genes and their regulatory elements.

**Fig. 1:**
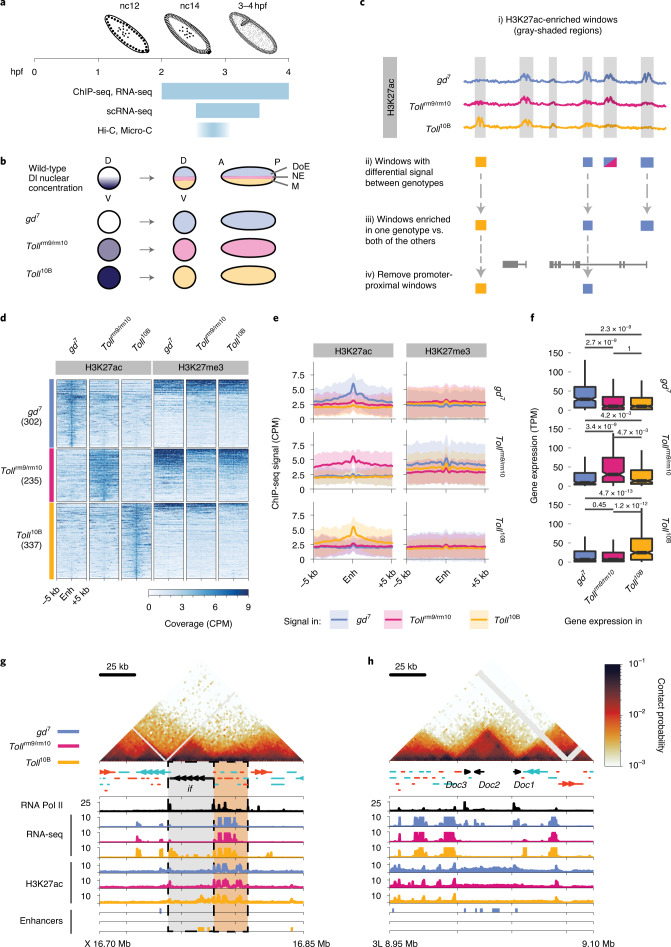
Identification of tissue-specific regulatory elements for dorsoventral patterning. **a**, Overview of early embryonic development in *Drosophila*. The syncytial blastoderm embryo undergoes 13 cycles of nuclear division before the maternal-to-zygotic transition occurs at nc14. This involves ZGA and embryo cellularization and is followed by gastrulation beginning around 3 hpf. ChIP-seq and RNA-seq datasets used in this study were derived from embryos at 2–4 hpf, including embryos at the late syncytial blastoderm stage, cellular blastoderm stage, gastrulation and the beginning of germ-band elongation. scRNA-seq datasets were derived from embryos at 2.5–3.5 hpf at the cellular blastoderm and gastrulation stages. Hi-C and Micro-C datasets were derived from hand-sorted cellular blastoderm embryos. **b**, Dorsoventral patterning of the *Drosophila* embryo is controlled by the nuclear concentration of Dl. High levels of nuclear Dl specify mesoderm (M, yellow), intermediate levels specify neuroectoderm (NE, pink), while nuclei without Dl become dorsal ectoderm (DoE, blue). The *gd*^7^, *Toll*^rm9/rm10^ and *Toll*^10B^ maternal effect mutations lead to embryos with uniform levels of nuclear Dl, which produce only dorsal ectoderm, neuroectoderm and mesoderm, respectively. D, dorsal; V, ventral; A, anterior; P, posterior. **c**, Schematic representation of the identification of putative tissue-specific enhancers using csaw^114,115^. We identified H3K27ac-enriched regions (gray-shaded areas) and performed pairwise comparisons between genotypes (*gd*^7^, blue; *Toll*^rm9/rm10^, pink; *Toll*^10B^, yellow) to identify differential H3K27ac levels between genotypes. Candidate tissue-specific enhancers are enriched for H3K27ac in one genotype compared to the other two genotypes and do not overlap a promoter. **d**, Heatmaps of H3K27ac and H3K27me3 ChIP-seq signal at putative tissue-specific enhancers (enh), normalized counts per million mapped reads (CPM) in 10-bp bins^116^. **e**, Average ChIP-seq signal for H3K27ac and H3K27me3 (CPM) at putative tissue-specific enhancers. Shaded areas represent ±1 s.d. from the mean. **f**, Expression of genes associated with putative tissue-specific enhancers. Top, *gd*^7^-specific enhancers; middle, *Toll*^*r*m9/rm10^-specific enhancers; bottom, *Toll*^10B^-specific enhancers. Box plots show median, box spans first to third quartiles, whiskers extend to smallest or largest values no further than 1.5× the interquartile range (IQR) from the box, and notches extend 1.58 × IQR × (√*n*)^−1^ from the median. Outliers are excluded for clarity. Two-sided Wilcoxon rank-sum test; *n* = 302 *gd*^7^ enhancer–gene pairs, *n* = 235 *Toll*^*r*m9/rm10^ enhancer–gene pairs, *n* = 337 *Toll*^10B^ enhancer–gene pairs. TPM, transcripts per million. **g**,**h**, Examples of chromatin organization at dorsoventral patterning genes (in black; *if*, **g**; *Doc1*, *Doc2*, *Doc3*, **h**). Top, normalized Hi-C contact probability at 2-kb resolution in control embryos at 3–4 hpf^50^. Positive-strand genes, orange; negative-strand genes, blue. Ser5-phosphorylated RNA Pol II ChIP-seq data (CPM) from control embryos, black^117^. RNA-seq and H3K27ac ChIP-seq data (CPM) are shown in blue (*gd*^7^), pink (*Toll*^*r*m9/rm10^) and yellow (*Toll*^10B^) (ref. ^62^, this study). Tissue-specific putative enhancers are indicated with color-coded bars. The gray-shaded area in **g** indicates a domain with a developmentally regulated gene; the orange-shaded region contains housekeeping genes.

Embryonic development requires precise regulation of gene expression, making it an ideal context in which to investigate the relationship between chromatin organization and gene regulation^46^. In particular, *Drosophila melanogaster* has long been used as a model organism for the study of development, and the key principles and factors involved in embryonic patterning are well understood^47,48^. Early *Drosophila* development involves a series of thirteen rapid, synchronous nuclear divisions, before the embryo becomes cellularized and undergoes zygotic genome activation (ZGA) at nuclear cycle (nc)14 (Fig. 1a)^49^. We and others previously showed that chromatin organization in *Drosophila* is established at nc14, coincident with ZGA^50,51^. While a small number of genes are zygotically expressed before the major wave of ZGA^52^, maternally provided cues are responsible for establishing the major anterior–posterior and dorsoventral axes^53,54^. Therefore, by ZGA, cells in different regions of the embryo contain different developmental transcription factors, have different patterns of chromatin accessibility^55,56^ and are primed to express different genes.

Cell fate along the dorsoventral axis is controlled by the nuclear concentration of the transcription factor Dorsal (Dl)^57,58^, which peaks during nc14 (ref. ^59^). Activation of the Toll signaling pathway on the ventral side of the embryo leads to high levels of Dl entering the nucleus, while Dl is excluded from the nucleus on the dorsal side^53^. Different levels of Dl concentration are responsible for the specification of different cell fates^57,58^ (Fig. 1b). Maternal effect mutations in the Toll pathway lead to uniform levels of Toll signaling across the whole embryo. Different mutations lead to different concentrations of nuclear Dl, making it possible to obtain females that produce a homogeneous population of embryos that consist entirely of presumptive mesoderm (*Toll*^10B^), neuroectoderm (*Toll*^rm9/rm10^) or dorsal ectoderm (*gd*^7^) (Fig. 1b). These embryos provide an excellent model system to study tissue-specific regulation during development, which has led to the discovery of key transcription factors, regulatory elements and processes required for embryo patterning^58,60–64^.

In this study, we use *Drosophila* dorsoventral patterning as a model system to investigate the relationship between tissue-specific gene regulation and 3D chromatin organization. We focus on the cellular blastoderm stage, approximately 2-3 h post-fertilization (hpf), which is coincident with nc14, establishment of chromatin organization and the onset of ZGA (Fig. 1a). At this stage, the presumptive dorsal ectoderm, neuroectoderm and mesoderm have been specified, but complex tissues have not been formed. We identify putative regulatory elements involved in dorsoventral patterning and show that their target genes are developmentally regulated and have a distinct chromatin organization compared to that of housekeeping genes. We find that, while there are clear differences in chromatin state and overall gene expression between embryos from *gd*^7^, *Toll*^rm9/rm10^ and *Toll*^10B^ mutant mothers (hereafter referred to as *gd*^7^, *Toll*^rm9/rm10^ and *Toll*^10B^ embryos), there is still substantial heterogeneity in gene expression at the single-cell level. However, these tissue-specific differences in chromatin state and gene expression are not associated with tissue-specific 3D chromatin organization. Together, these results provide evidence that tissue-specific chromatin conformation is not required for tissue-specific gene expression. Rather, our findings indicate that the organization of the genome into 3D chromatin domains acts as an architectural framework to facilitate correct regulation of gene expression once enhancers become active.

## Results

### Identification of regulatory elements and genes involved in dorsoventral patterning

**Extended Data Fig. 1:**
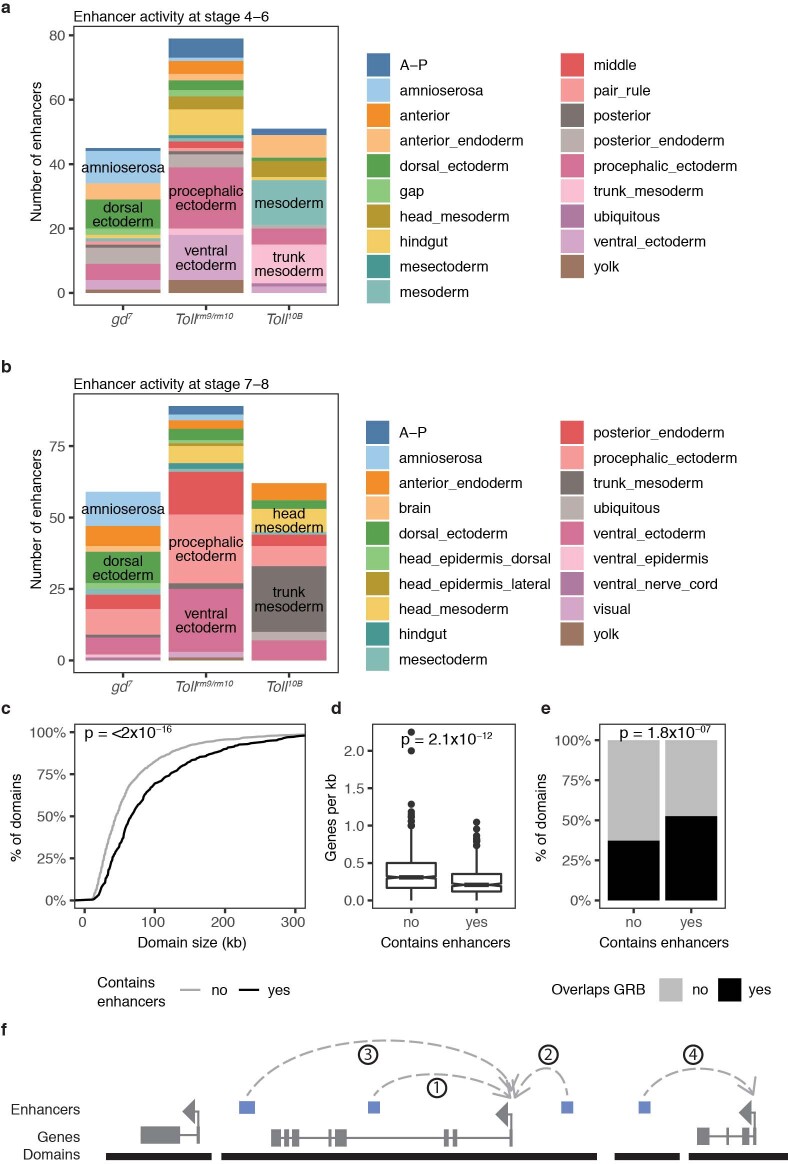
Properties of domains containing tissue-specific putative enhancers and validation of tissue-specific enhancer activity. **a, b**, Putative enhancers active in *gd*^*7*^, *Toll*^*rm9/rm10*^, and *Toll*^*10B*^ embryos overlap with enhancers from ref. ^131^ and are enriched for enhancers that drive expression in relevant regions of the embryo at stages 4-6 (**a**) and 7-8 (**b**). The two largest categories are labelled for each stacked bar. **c**, Size of domains containing putative tissue-specific enhancers (n = 394 domains, black) and those without enhancers (n = 1097 domains, grey). Two-sided Wilcoxon rank sum test, p < 2×10^-16^. **d**, Genes per kilobase inside domains containing putative tissue-specific enhancers (n = 394 domains) and those without enhancers (n = 1097 domains). Two-sided Wilcoxon rank sum test, p = 2.1 ×10^-12^. Box plots show median, box spanning first to third quartiles, whiskers extending to smallest/largest values no further than 1.5 * IQR from the box, and notches extending 1.58 * IQR / sqrt(n) from the median. Outliers are excluded for clarity. **e**, Overlap of chromatin domains (n = 1491 domains) with Genomic Regulatory Blocks (GRBs) from ref. ^14^. Chi-squared test p = 1.8 ×10^-7^. **f**, Schematic illustration of assignment of enhancers to target genes. Broadly expressed and non-expressed genes are omitted for clarity (see Methods). Enhancers are assigned to genes as follows: (1) enhancers that overlap a transcript are assigned to that gene; (2) remaining unassigned enhancers are assigned to the closest promoter, unless that promoter is in a different domain; (3) remaining enhancers are assigned to the closest promoter in the same domain; (4) remaining enhancers are assigned to the closest promoter, even if it is in a different domain.

To understand the relationship between tissue-specific gene regulation and genome organization during embryonic development, we first sought to identify a stringent genome-wide set of candidate tissue-specific regulatory elements involved in dorsoventral patterning. We performed chromatin immunoprecipitation sequencing (ChIP-seq) for histone 3 lysine 27 acetylation (H3K27ac), associated with active chromatin, and trimethylation of H3K27(me3), associated with repression, in *Toll*^rm9/rm10^ embryos at 2–4 hpf and combined this with ChIP-seq data from *gd*^7^ and *Toll*^*10B*^ embryos at 2–4 hpf from ref. ^62^. Embryos collected at 2–4 hpf largely consist of embryos in the late cellular blastoderm stage and embryos undergoing gastrulation, thus targeting our time point of interest. Note that, while H3K27ac is established from nc12 onwards, H3K27me3 is only present from mid-nc14 (ref. ^65^). Using these data, we carried out genome-wide differential peak identification for H3K27ac (Fig. 1c). We identified 302 regions enriched for H3K27ac in *gd*^7^ embryos compared to those in both *Toll*^rm9/rm10^ and *Toll*^10B^ embryos, 235 regions specifically enriched in *Toll*^rm9/rm10^ embryos and 337 regions specifically enriched in *Toll*^10B^ embryos (Fig. 1d). By requiring significant enrichment in one genotype compared to both of the other genotypes, we selected a highly stringent set of regions with tissue-specific increases in H3K27ac. These putative enhancers overlap with genomic regions that were shown to drive expression in the expected regions of the embryo (Extended Data Fig. 1d,e). The putative enhancers were also depleted of the repressive chromatin mark H3K27me3 in the genotypes in which they were enriched for H3K27ac, compared to the other genotypes (Fig. 1d,e), providing further evidence for their tissue specificity. Next, we assigned putative enhancers to target genes, using a combination of gene expression data, linear genomic proximity and chromatin conformation data (Methods and Supplementary Table 1), and verified that genes assigned to tissue-specific candidate enhancers had significantly higher expression in the tissue where the enhancer was active (Fig. 1f). We conclude that the identified regions represent a stringent set of candidate enhancers associated with the regulation of dorsoventral patterning.

### Developmentally regulated genes have a distinct regulatory landscape compared to that of housekeeping genes

**Extended Data Fig. 2:**
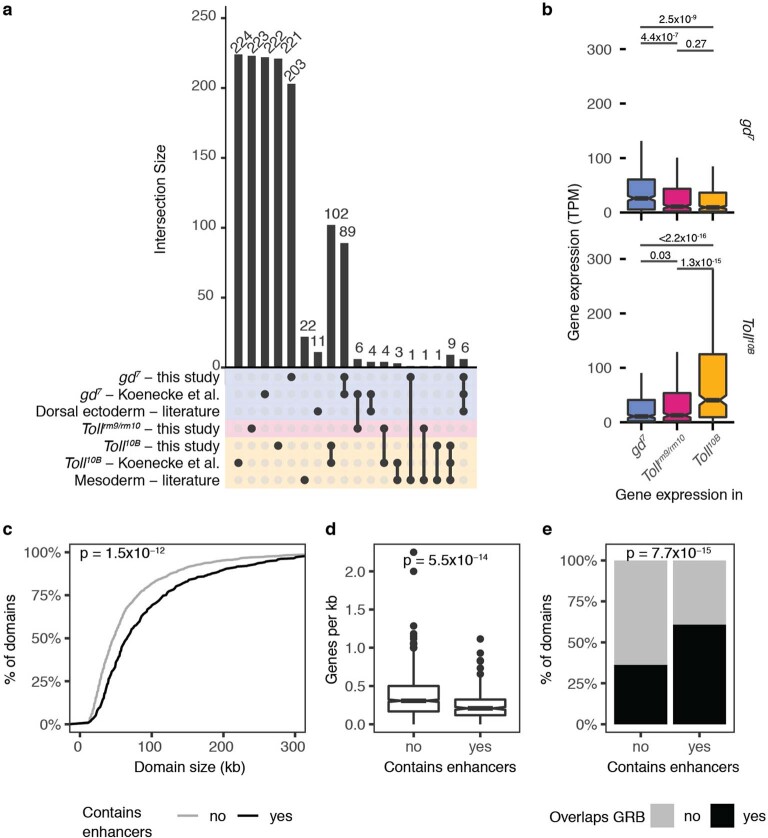
Properties of domains containing tissue-specific putative enhancers are robust to different enhancer definitions. **a**, UpSet plot^132,133^ showing overlap between the putative tissue specific enhancers identified in this study, in ref. ^62^, and mesoderm and dorsal ectoderm enhancers identified from a literature search by^62^. Lower panel y axis represents enhancer sets; intersections between these sets are shown by joined dots. Bars in upper panel represent intersection sizes. For example, 6 putative dorsal ectoderm enhancers are identified in this study, identified in^62^, and also identified from a literature search (rightmost column). **b**, Expression of genes associated with putative enhancers identified by^62^ in *gd*^*7*^ and *Toll*^*10B*^ mutant embryos. Top, genes associated with *gd*^*7*^-specific putative enhancers (n = 380 enhancer-gene pairs); bottom, genes associated with *Toll*^*10B*^-specific putative enhancers (n = 416 enhancer-gene pairs). Box plots show median, box spanning first to third quartiles, whiskers extending to smallest/largest values no further than 1.5 * IQR from the box, and notches extending 1.58 * IQR / sqrt(n) from the median. Outliers are excluded for clarity. **c**, Size of domains containing putative tissue-specific enhancers identified by^62^ (n = 313 domains, black) and those without enhancers (n = 1178 domains, grey). Two-sided Wilcoxon rank sum test, p = 1.5 ×10^-12^. **d**, Genes per kilobase inside domains containing putative tissue-specific enhancers (n = 313 domains) identified by^62^ and those without enhancers (n = 1178 domains). Two-sided Wilcoxon rank sum test p = 5.5 ×10^-14^. Box plots show median, box spanning first to third quartiles, whiskers extending to smallest/largest values no further than 1.5 * IQR from the box, and notches extending 1.58 * IQR / sqrt(n) from the median. **e**, Overlap of chromatin domains (n = 1491 domains) with Genomic Regulatory Blocks (GRBs) from ref. ^14^. Chi-squared test p = 7.7 ×10^-15^.

We next assessed the chromatin conformation landscape around these tissue-specific regulatory elements and their target genes. Using whole-genome chromosome conformation capture (Hi-C) data from *Drosophila* embryos at 3–4 hpf^50^, we observed that dorsoventral patterning genes were located in self-interacting domains, along with their assigned regulatory elements (for example, Fig. 1g, gray-shaded region, and Fig. 1h). These domains were larger than domains not associated with developmentally regulated genes (Extended Data Fig. 1a,b) (mean size of 94 kb, compared to 66 kb, *P* < 2.22 × 10^−16^). This was in contrast to housekeeping genes, which were enriched at the boundaries between domains and in small domains^50,66,67^ (Fig. 1g, orange-shaded region). In addition, domains containing developmentally regulated genes were significantly more likely to overlap with the large regions of high non-coding sequence conservation known as genomic regulatory blocks^14,68,69^ (Extended Data Fig. 1c). These results were robust for different definitions of tissue-specific enhancers (from ref. ^62^; Extended Data Fig. 2) and emphasize the distinct organization of developmentally regulated and housekeeping genes in the *Drosophila* genome.

### Single-cell expression analysis reveals heterogeneity in gene expression in dorsoventral mutant embryos

**Fig. 2:**
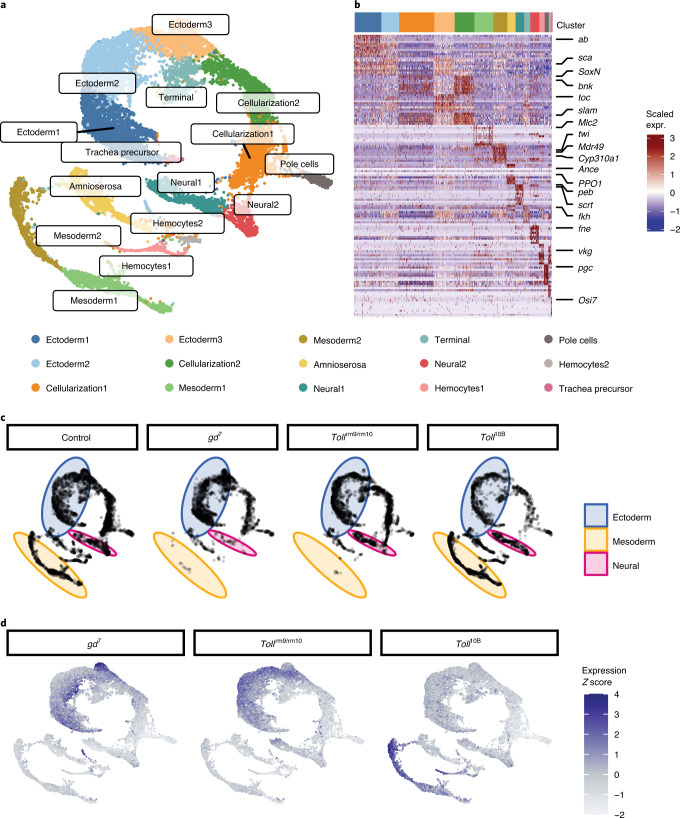
scRNA-seq analysis of gene expression during dorsoventral patterning. **a**, Clustering of single-cell gene expression (scRNA-seq) profiles from embryos at 2.5–3.5 hpf reveals clusters corresponding to distinct cell populations. **b**, Expression (expr.) of cluster marker genes (rows) in single cells (columns) from control embryos. The top ten marker genes for each cluster are shown, based on log_2_ fold change of expression within the cluster compared to outside the cluster. Selected marker genes are labeled. **c**, Single-cell gene expression profiles separated by cell origin. Certain clusters are depleted in the dorsoventral mutant embryos. Blue color indicates the area of the graph corresponding to ectoderm clusters; pink corresponds to neural; yellow corresponds to mesoderm. **d**, Single-cell expression of genes associated with putative tissue-specific enhancers. Color represents the *Z* score of average expression of genes associated with each group of tissue-specific enhancers.

**Extended Data Fig. 3:**
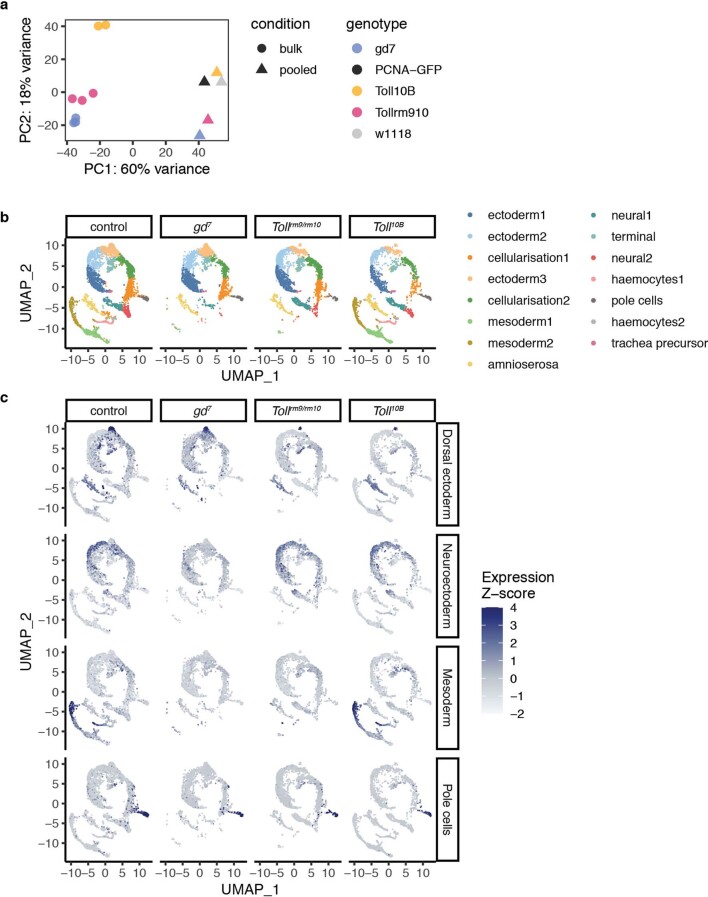
Validation of scRNA-seq data. **a**, PCA of pooled single-cell RNA-seq data and RNA-seq data. The first principal component separates techniques while the second principal component separates genotypes. Replicate control single-cell RNA-seq experiments cluster together, demonstrating robustness. **b**, Clustering of single-cell gene expression profiles from 2.5-3.5 hpf embryos reveals clusters corresponding to distinct cell populations, as in Fig. 2a but separated by genotype of origin. **c**, Expression of tissue-specific marker genes across single cells from different genotype origins. Marker genes for dorsal ectoderm (*Ance, CG2162, Doc1, Doc2, egr, peb, tok, ush, zen*), neuroectoderm (*ac, brk, CG8312, l(1)sc, mfas, Ptp4E, sog, SoxN, vnd*), mesoderm (*CG9005, Cyp310a1, GEFmeso, ltl, Mdr49, Mes2, NetA, ry, sna, stumps, twi, wgn, zfh1*), and pole cells (*pgc*) were obtained from^71^.

While the *gd*^7^, *Toll*^rm9/rm10^ and *Toll*^10B^ maternal effect mutants have long been used as models to analyze tissue-specific regulation during dorsoventral patterning^58,60,61^, the extent of cell fate conversion at the single-cell level in these embryos is unknown. Anterior–posterior patterning mechanisms are still active, and RNA in situ hybridization experiments suggest that cell fate conversion may be incomplete^57^. To assess the heterogeneity of gene expression and cell identities in these embryos, we carried out single-cell gene expression analysis using the 10x Genomics Chromium platform, analyzing a total of 16,790 cells with high-quality data across all genotypes. We used embryos at 2.5–3.5 hpf to target the late cellular blastoderm stage when dorsoventral patterning has been established (Fig. 1a)^59,70^. Clustering of single-cell expression profiles from wild-type and mutant embryos showed good concordance with bulk RNA-seq data (Extended Data Fig. 3a) and identified 15 clusters representing different cell identities in the embryo (Fig. 2a,b and Extended Data Fig. 3c). We identified upregulated marker genes in each cluster and used these to identify the cell identities in these clusters. We identified clusters representing mesoderm (*twi*, *Mlc2*, *Mdr49*) and ectoderm (*ab*, *sca*, *SoxN*), in addition to other cell populations, such as amnioserosa (*Ance*, *peb*), terminal regions of the embryo (*fkh*), pole cells (*pgc*), hemocytes (*PPO1*) and trachea precursors (Osiris gene cluster). Two clusters express cellularization stage genes (*bnk*, *slam*) and represent cells from embryos in the earlier stages of cellularization due to the timed collection. A full list of clusters and cluster marker genes is available in Supplementary Table 2.

Visualization of single-cell gene expression profiles from *gd*^7^, *Toll*^rm9/rm10^ and *Toll*^10B^ embryos revealed that specific clusters were depleted in these mutant embryos (Fig. 2c and Extended Data Fig. 3b,c). Cells from clusters representing mesoderm cell fates were almost completely absent in *gd*^7^ and *Toll*^rm9/rm10^ embryos, and subsets of ectoderm cells were missing in each of the mutants (Fig. 2c and Extended Data Fig. 3b,c). To further dissect the ectoderm clusters and identify cells corresponding to dorsal ectoderm and neuroectoderm, we visualized the expression of genes assigned to tissue-specific enhancers (Fig. 2d) and known dorsal ectoderm, neuroectoderm and mesoderm marker genes (Extended Data Fig. 3c; ref. ^71^). This revealed that the ectoderm clusters contained distinct subpopulations of cells expressing dorsal ectoderm markers and neuroectoderm markers. These subpopulations corresponded to the regions of the cell distribution that were depleted in *Toll*^rm9/rm10^ and *gd*^7^ embryos, respectively (Fig. 2c, compare distributions in the ‘ectoderm’ region). Despite the substantial level of cell-to-cell heterogeneity found in the mutant embryos, we observed that certain cell fates were lost. Importantly, the loss of specific cell fates combined with the tissue-specific enhancer usage shown above supports the use of these embryos to model dorsoventral patterning perturbations.

### Major features of chromatin organization are maintained across tissues

We next asked how differential usage of regulatory elements and differential gene expression relate to chromatin conformation during dorsoventral patterning. To do so, we generated Hi-C datasets for *gd*^7^, *Toll*^rm9/rm10^, *Toll*^10B^ and control embryos at the cellular blastoderm stage (late nc14, approximately 2.5–3 hpf) at 2-kb resolution (Supplementary Table 3).

**Fig. 3:**
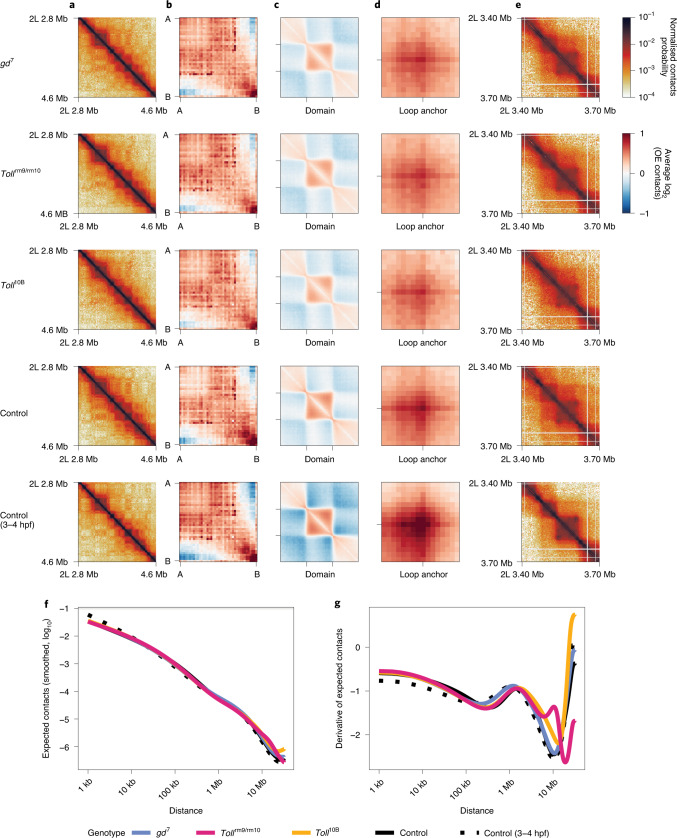
Global chromatin conformation along the dorsoventral axis. **a**, Chromatin conformation for a 1.8-Mb region of chromosome 2L in *gd*^7^, *Toll*^*r*m9/rm10^, *Toll*^10B^ and control embryos at the cellular blastoderm stage and control embryos at 3–4 hpf^50^. **b**. ‘Saddle plot’ representing genome-wide average chromatin compartmentalization. Active regions interact with other active regions (top left), while inactive regions interact with other inactive regions (bottom right). **c**, Aggregate analysis of domains identified using Hi-C data from embryos at 3–4 hpf at 2-kb resolution. **d**, Aggregate analysis of chromatin loops identified in ref. ^72^. **e**, Chromatin conformation for a 300-kb region of chromosome 2L. OE, observed/expected. **f**, Average contact probability by distance for control (black), *gd*^7^ (blue), *Toll*^rm9/rm10^ (pink) and *Toll*^10B^ (yellow) embryos. **g**, The derivative of the expected contact probability by distance, highlighting differences between samples at far-cis distances due to the presence of rearranged balancer chromosomes in *Toll*^rm9/rm10^ and *Toll*^10B^ embryos.

**Extended Data Fig. 4:**
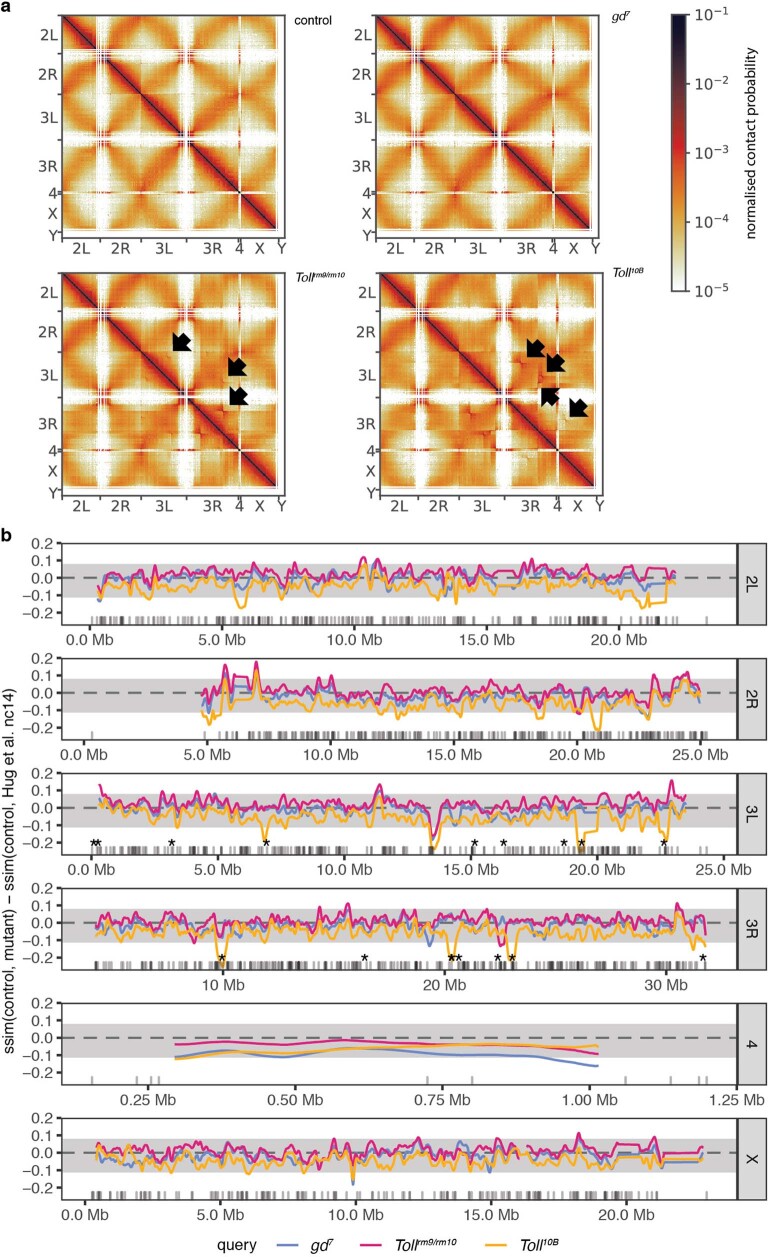
Balancer chromosomes contribute to differences between Hi-C matrices in embryos from different genotypes. **a**, Whole genome contact probability maps for control, *gd*^*7*^, *Toll*^*rm9rm/10*^, and *Toll*^*10B*^ embryos at the cellular blastoderm stage. Arrows mark artefacts in the Hi-C data that indicate rearrangements on balancer chromosomes present in a subset of *Toll*^*rm9rm/10*^ (TM6) and *Toll*^*10B*^ (TM3 and OR60) embryos. **b**, CHESS^75^ similarity scores were calculated between mutant and control embryo Hi-C datasets (5 kb resolution, 500 kb window size). As a reference, similarity scores were calculated between control embryo Hi-C data from this study and Hug et al. ^50^. The difference between the reference similarity score and the control/mutant similarity score is shown for all chromosomes (blue, *gd*^*7*^; pink, *Toll*^*rm9/rm10*^; yellow, *Toll*^*10B*^). Values around zero represent regions where chromatin conformation is similar between control and mutant, while negative values represent regions where there are greater differences between control and mutant than between the reference control datasets. Shaded area, +/- two standard deviations from genome-wide mean. Grey ticks, positions of genes that are differentially expressed between dorsoventral mutant embryos^62^. Asterisks mark positions of known rearrangement breakpoints on the TM3 and TM6 balancer chromosomes^35,118^.

Systematic comparison of the Hi-C datasets across genotypes revealed that, on average, characteristic features of chromatin conformation were similar across datasets (Fig. 3). Saddle plots revealed similar strength of compartmentalization in control and *gd*^7^, *Toll*^rm9/rm10^ and *Toll*^10B^ embryos (Fig. 3a,b). We next analyzed overall self-interacting domain strength using domains identified in control embryos at 3–4 hpf as a reference (Fig. 3c). While domain strength was weaker in cellular blastoderm embryos than that in embryos at 3–4 hpf, the strength was similar across all genotypes, suggesting that the vast majority of domains and domain boundaries were present in all tissues. We obtained similar conclusions when we examined chromatin loop strengths, using loops from Kc167 cells^72^ as a reference (Fig. 3d,e), indicating that loops were maintained across tissues. Finally, we analyzed genome-wide contact probability decay with distance (*P*(*s*)). A shallow slope at distances <100 kb reflects local chromatin compaction into domains, while the flattening of the slope around separation distances of 1 Mb indicates compartment formation (refs. ^33,73,74^; Fig. 3f). We also examined the derivative of *P*(*s*), as this can highlight differences in the strength of domain formation (Fig. 3g)^74^. These analyses revealed differences in these profiles at distances >5 Mb, which corresponded to genomic rearrangements on balancer chromosomes present in a subset of *Toll*^rm9/rm10^ and *Toll*^10B^ embryos (Extended Data Fig. 4). Combined, our results demonstrate that overall genome organization at the level of compartments, domains and chromatin loops is highly similar across genotypes, suggesting that it is maintained across tissues in cellular blastoderm embryos.

### Chromatin conformation at developmentally regulated genes is similar across tissues, despite differences in gene expression and chromatin state

**Fig. 4:**
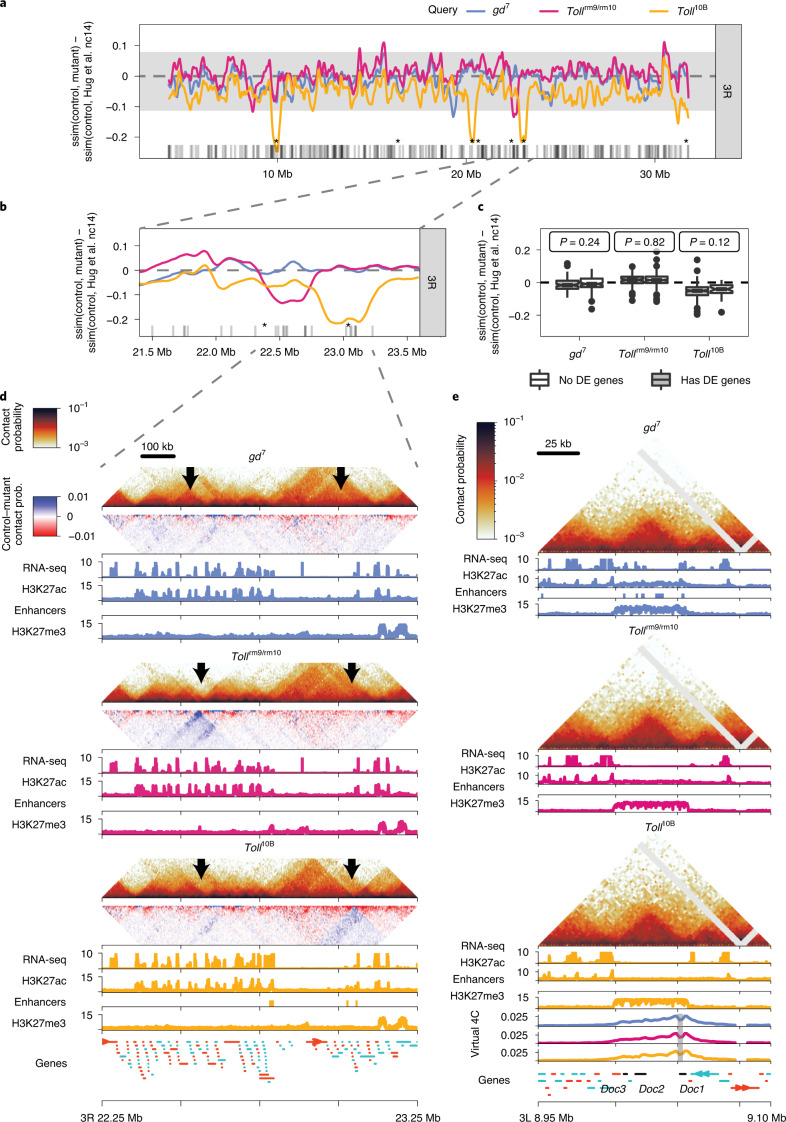
Chromatin conformation is not affected by tissue-specific gene expression. **a**, CHESS^75^ similarity scores (structural similarity index, ssim) were calculated between mutant and control embryo Hi-C datasets (5-kb resolution, 500-kb window size). As a reference, similarity scores were calculated between control embryo Hi-C data from this study and ref. ^50^. The difference between reference ssim and control–mutant ssim is shown for chromosome 3R (blue, *gd*^7^; pink, *Toll*^rm9/rm10^; yellow, *Toll*^10B^). Values around zero indicate similar chromatin conformation in control and mutant embryos, while negative values indicate greater differences between control and mutant embryos than between the reference control datasets^50^. Shaded area, ±2 s.d. from genome-wide mean. Gray ticks indicate DE genes in dorsoventral mutant embryos^62^. Asterisks indicate known breakpoint positions for balancer chromosomes present in a subset of *Toll*^rm9/rm10^ and *Toll*^10B^ embryos (TM6 and TM3, respectively^35,118^). **b**, Example of ssim differences for a 2-Mb region on chromosome 3R. Low ssim differences correlate with the positions of known breakpoints (asterisks) rather than with DE genes. Hi-C data for a subset of this region are shown in **d**. **c**, Box plots of ssim differences for genomic windows with and without genes that were DE between the indicated genotype and both of the other genotypes^62^. Since adjacent windows overlap, every hundredth window was selected to obtain a non-overlapping set of windows (*n* = 101 genomic windows with DE genes and 124 windows without DE genes for *gd*^7^ embryos; *n* = 74 windows with DE genes and 152 windows without DE genes for *Toll*^rm9/rm10^ embryos; *n* = 183 windows with DE genes and 41 windows without DE genes for *Toll*^10B^ embryos). *P* values were determined by two-sided Wilcoxon rank-sum test. Box plots show median, box spanning first to third quartiles, whiskers extending to smallest or largest values no further than 1.5 × IQR from the box and notches extending 1.58 × IQR × (√*n*)^−1^ from the median. **d**, Tissue-specific chromatin data for a 1-Mb subset of the region on chromosome 3R shown in **b** above. For each genotype, top, normalized Hi-C contact probability (prob.) and contact probability difference at 5-kb resolution; red, increased contact probability in embryos of the mutant genotype; blue, decreased contact probability. Arrows indicate changes in chromatin conformation in *Toll*^rm9/rm10^ and *Toll*^10B^ embryos at known balancer chromosome breakpoints. Bottom, RNA-seq (CPM)^62^ and H3K27ac and H3K27me3 ChIP-seq data (CPM^62^, this study). Tissue-specific putative enhancers are indicated by color-coded bars beneath the corresponding H3K27ac ChIP-seq track. Lower panel: positive-strand genes, orange; negative-strand genes, blue. See also additional example loci in Extended Data Fig. 5. **e**, Tissue-specific chromatin conformation data for a 150-kb region around the *Doc1*, *Doc2* and *Doc3* genes. For each genotype, normalized Hi-C contact probability at 2-kb resolution (top), RNA-seq data (CPM)^62^ (middle), H3K27ac and H3K27me3 ChIP-seq data (CPM^62^, this study) (bottom). Tissue-specific putative enhancers are indicated by color-coded bars beneath the corresponding H3K27ac ChIP-seq track. Bottom, ‘virtual 4C’ tracks, representing interactions of a 2-kb region around the promoter of *Doc1*. Positive-strand genes, orange; negative-strand genes, blue. See also additional example loci in Extended Data Fig. 6.

**Extended Data Fig. 5:**
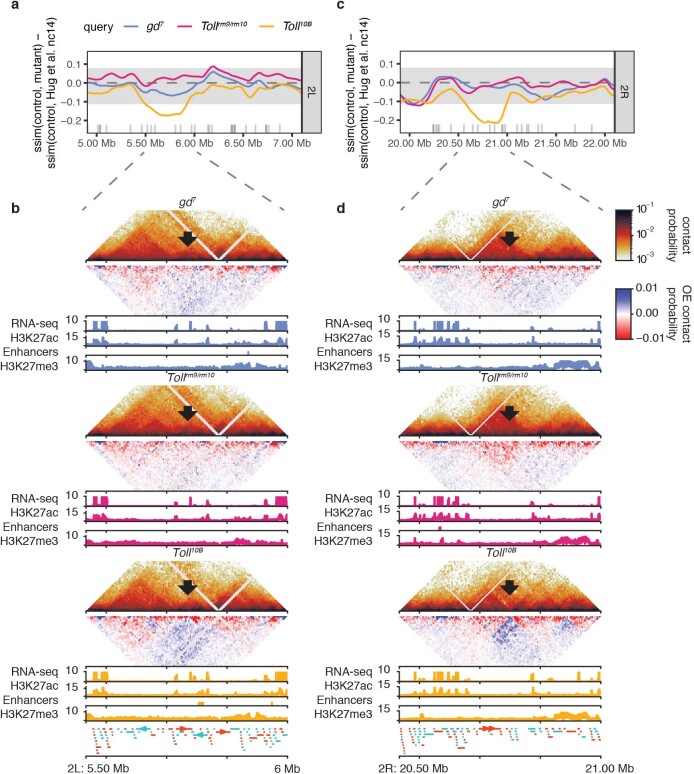
A small number of regions have changes in chromatin conformation detected by CHESS that are not associated with known genomic rearrangements. **a, c**, CHESS^75^ similarity scores were calculated between mutant and control embryo Hi-C datasets (using 5 kb resolution, 500 kb window size). As a reference, similarity scores were calculated between control embryo Hi-C data from this study and Hug et al. ^50^. The difference between this reference similarity score and the control/mutant similarity score is shown (blue, *gd*^*7*^; pink, *Toll*^*rm9/rm10*^; yellow, *Toll*^*10B*^). Values around zero represent regions where chromatin conformation is similar between control and mutant, while negative values represent regions where there are greater differences between control and mutant than between the reference control datasets. Shaded area, +/- two standard deviations from genome-wide mean. Grey ticks, positions of genes that are differentially expressed between dorsoventral mutant embryos^62^. **b, d**, For each genotype, top, normalized Hi-C contact probability and contact probability difference (5 kb resolution); red, regions with increased contact probability in embryos of the mutant genotype; blue, decreased contact probability. Arrows, regions with a change in contact probability in *Toll*^*10B*^. Bottom, RNA-seq (CPM)^62^, H3K27ac and H3K27me3 ChIP-seq data (CPM^62^, this study). Tissue-specific putative enhancers, color-coded bars beneath corresponding H3K27ac ChIP-seq track. Lower panel, gene annotations. Positive-strand genes, orange; negative strand genes, blue. **a**, Example of differences in CHESS similarity scores for a 2 Mb region on chromosome 2 L. **b**, Hi-C data for a 500 kb subset of the region shown in **a. c**, Example of differences in CHESS similarity scores for a 2 Mb region on chromosome 2 R. **d**, Hi-C data for a 500 kb subset of the region shown in **c**.

**Extended Data Fig. 6:**
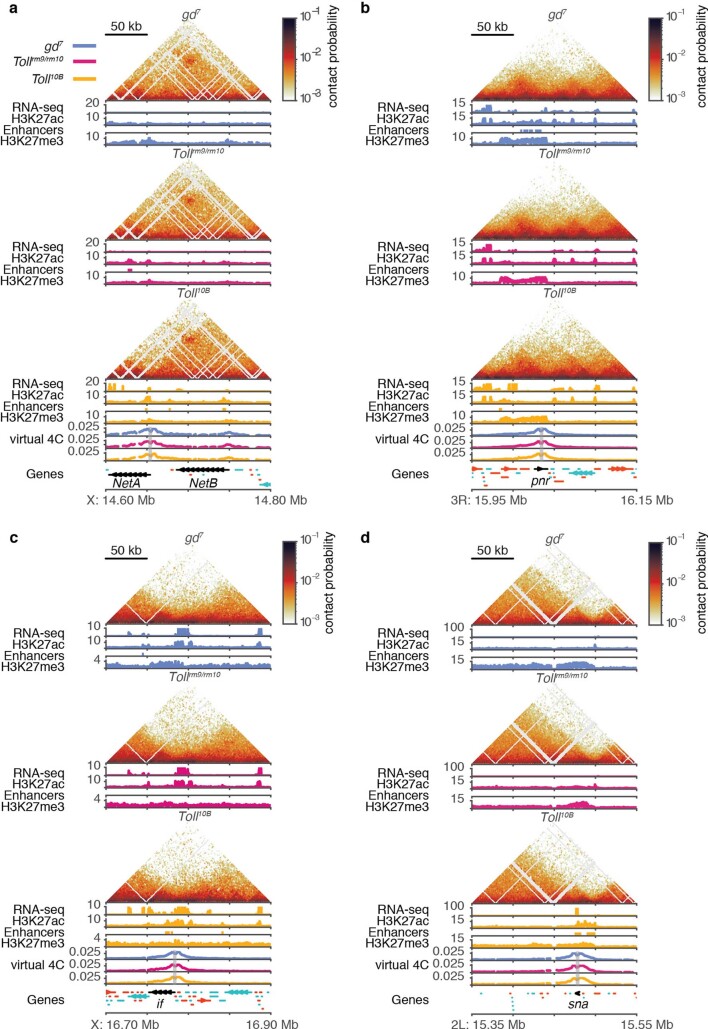
Chromatin conformation is not affected by tissue-specific gene expression. Tissue-specific chromatin data for regions containing dorsoventral patterning genes. For each genotype, top, normalized Hi-C contact probability (2 kb resolution); middle, RNA-seq data (CPM)^62^; bottom, H3K27ac and H3K27me3 ChIP-seq data (CPM^62^, this study). Tissue-specific putative enhancers, color-coded bars beneath corresponding H3K27ac ChIP-seq track. Lower panels, ‘virtual 4 C’ tracks for each genotype representing interactions of a 2 kb region around the promoters of genes of interest, as highlighted by the grey rectangle; gene annotations. Positive-strand genes, orange; negative strand genes, blue. Dorsoventral patterning genes of interest, black. **a**, *NetA* and *NetB*. **b**, *pnr*. **c**. *if*. **d**, *sna*.

To systematically assess chromatin conformation across the genome and identify regions with differences, we used Comparison of Hi-C Experiments using Structural Similarity (CHESS)^75^, an approach for differential chromatin conformation detection based on computer vision techniques. Briefly, Hi-C submatrices are compared genome-wide between pairs of datasets to produce a similarity score and a signal-to-noise ratio for each pair of genomic windows (Methods). Using this approach, we compared control and mutant embryos at the cellular blastoderm stage at 5-kb resolution and with a 500-kb window size. As a reference, we compared control cellular blastoderm stage data from this study with Hi-C data from nc14 embryos from ref. ^50^. Subtracting this reference score from the score for each control–mutant comparison allowed us to identify regions with specific differences in genome organization between control and mutant embryos and exclude regions with low similarity scores due to noise. This analysis revealed that most regions across the genome did not display significant differences in 3D chromatin organization between *gd*^7^, *Toll*^rm9/rm10^ and *Toll*^10B^ embryos (Fig. 4a,f and Extended Data Figs. 4 and 5). This agreed with visual examinations of control–mutant difference matrices (Fig. 4d). The subset of regions that did display strong changes in chromatin organization between genotypes could be attributed to genomic rearrangements present on balancer chromosomes in a subset of the *Toll*^rm9/rm10^ and *Toll*^10B^ embryos, rather than correlating with the locations of genes that were differentially expressed (DE) (Fig. 4a,b,d and Extended Data Figs. 4–6). Of two additional example loci on chromosome 2 with changes in chromatin organization identified by CHESS in *Toll*^10B^ embryos, one was close to a cluster of *Toll*^10B^-specific enhancers (Extended Data Fig. 5b), while the other occurred in a region devoid of H3K27ac, H3K27me3 or RNA-seq signal in any of the mutant genotypes (Extended Data Fig. 5d). To further investigate the relationship between changes in chromatin conformation and gene expression, we analyzed CHESS similarity scores in windows containing genes that were DE in the mutant embryos^62^ compared to those in other genomic windows (Fig. 4c). This revealed a lack of association between differential gene expression and differential chromatin structure at the genome-wide level.

**Extended Data Fig. 7:**
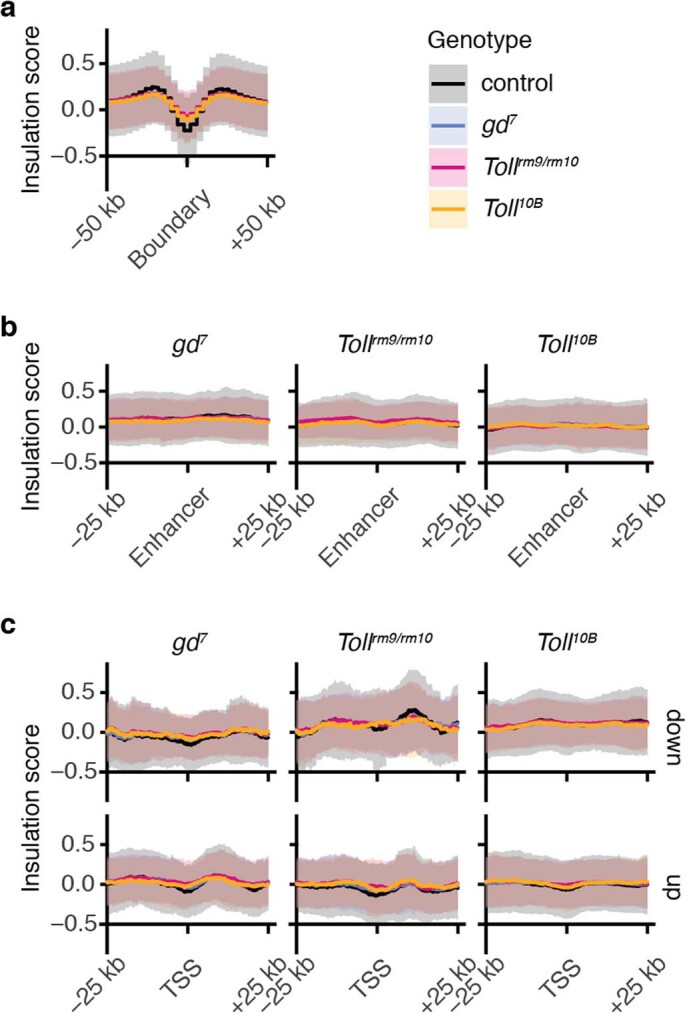
Insulation does not change across genotypes at tissue-specific enhancers or differentially expressed genes. **a**, Average insulation scores around consensus boundaries identified in 3-4 hpf Hi-C^50^. Insulation scores from control, *gd*^*7*^, *Toll*^*rm9/rm10*^, and *Toll*^*10B*^ embryos at the cellular blastoderm stage were calculated at 2 kb resolution with a 16 kb window size. Shaded areas represent +/− one standard deviation from the mean. **b**, Average insulation scores around *gd*^*7*^, *Toll*^*rm9/rm10*^, and *Toll*^*10B*^ -specific enhancers. **c**, Average insulation scores around the transcription start sites (TSSs) of genes that are up- or down-regulated in the given genotype compared to both other genotypes.

To further validate these observations, we visually assessed genome organization in *gd*^7^, *Toll*^rm9/rm10^ and *Toll*^10B^ embryos at known DE dorsoventral patterning genes (Fig. 4e and Extended Data Fig. 6). Examination of chromatin conformation and chromatin state data at these regions did not indicate any differences in domain organization, boundary formation or loop formation. For example, the *Doc1*, *Doc2* and *Doc3* genes, which encode T-box transcription factors that are specifically expressed in *gd*^7^ embryos and are required for amnioserosa differentiation and dorsolateral ectoderm patterning^76^, lie in a well-insulated domain that contained multiple *gd*^7^-specific putative enhancers and was enriched for H3K27me3 in all three mutants at 2–4 hpf, although to a lesser extent in *gd*^7^ embryos (Fig. 4e). There was no evidence of changes in the insulation of this domain in *gd*^7^ embryos, in which the genes were active, compared to those in the other datasets, nor was there any change in its internal structure, such as changes in interactions between enhancers and the target gene promoters. Similar conclusions were obtained by examining additional loci, including the *pnr* locus (expressed in *gd*^7^ embryos) and the *NetA*, *NetB*, *if* and *sna* loci, which are active in *Toll*^10B^ embryos (Extended Data Fig. 6). To further investigate the effects of tissue-specific enhancer activity and gene expression on chromatin organization, we examined insulation scores^77^. Average insulation score was low at domain boundaries (Extended Data Fig. 7a) but did not show local decreases at enhancers or the transcription start sites of DE genes. Importantly, average insulation scores did not change across genotypes (Extended Data Fig. 7), providing further evidence for the maintenance of chromatin conformation. Together, these results suggest that tissue-specific gene expression and enhancer activity do not necessarily involve changes in domain organization.

**Extended Data Fig. 8:**
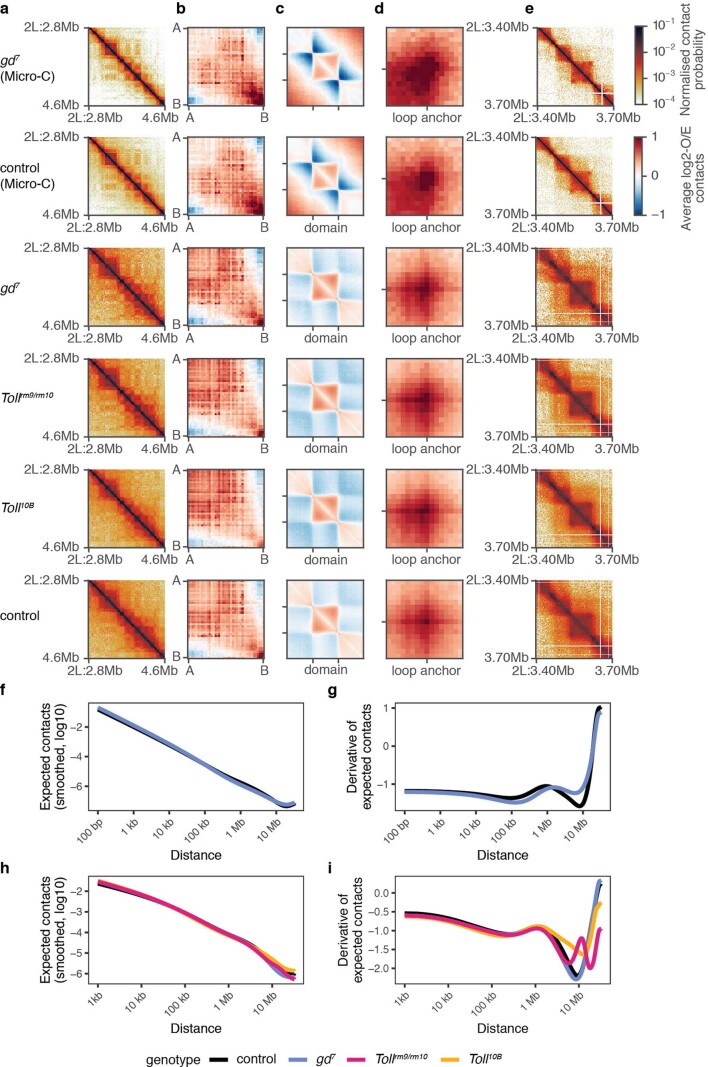
Global chromatin conformation along the dorsoventral axis by Micro-C at the cellular blastoderm stage and Hi-C at stage 10. Chromatin conformation for a 1.8 Mb region of chromosome 2 L by Micro-C in control and *gd*^*7*^ embryos at the cellular blastoderm stage and *gd*^*7*^, *Toll*^*rm9/rm10*^, *Toll*^*10B*^, and by Hi-C in control embryos at stage 10. **b**, ‘Saddle-plot’ representing genome-wide average chromatin compartmentalisation. Active regions tend to interact with other active regions (top left), while inactive regions interact with other inactive regions (bottom right). **c**, Aggregate analysis of domains identified using Hi-C data from 3-4 hpf embryos at 2 kb resolution. **d**, Aggregate analysis of chromatin loops identified in^72^. **e**, Chromatin conformation for a 300 kb region of chromosome 2 L, showing domains and a loop. **f**, Average Micro-C contact probability at different distances for control (black) and *gd*^*7*^ (blue) embryos at the cellular blastoderm stage. **g**, The derivative of the expected Micro-C contact probability by distance. **h**, Average Hi-C contact probability at different distances for control (black), *gd*^*7*^ (blue), *Toll*^*rm9/rm10*^ (pink), and *Toll*^*10B*^ (yellow) embryos at stage 10. **i**, The derivative of the expected Hi-C contact probability by distance for stage 10 embryos, highlighting differences between samples at far-cis distances due to the presence of balancer chromosomes in the *Toll*^*rm9/rm10*^ and *Toll*^*10B*^ embryos.

**Extended Data Fig. 9:**
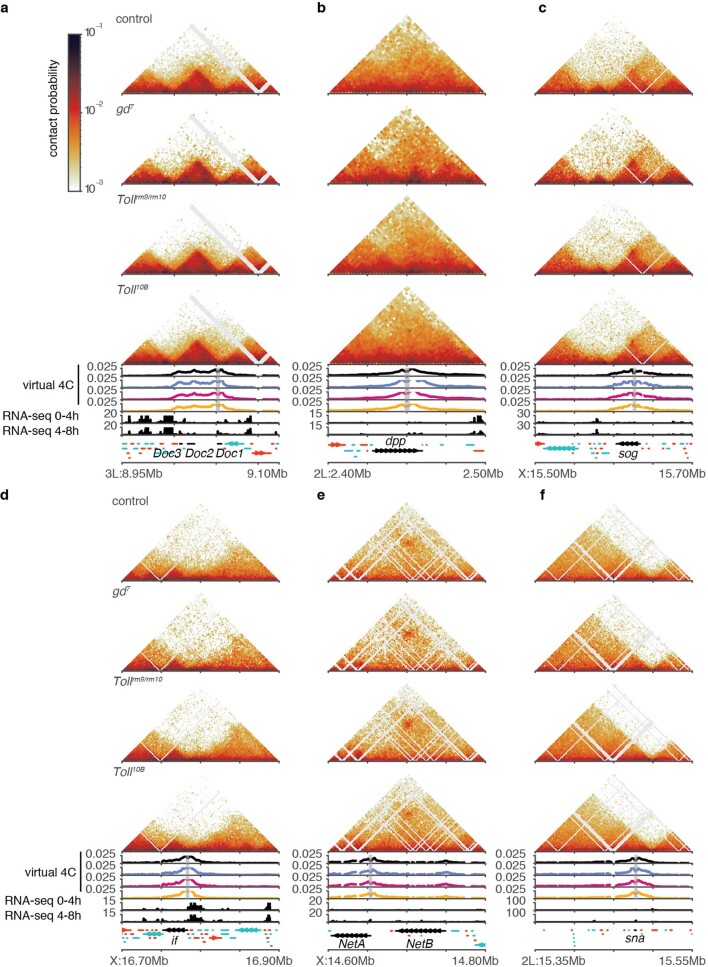
Chromatin conformation remains similar across tissues at a later developmental stage. Tissue-specific Hi-C data from stage 10 embryos (approximately 4-5 hpf) for regions containing dorsoventral patterning genes. For each genotype, top, normalized Hi-C contact probability, 2 kb resolution. Middle, ‘virtual 4 C’ tracks for each genotype representing interactions of a 2 kb region around the promoters of genes of interest (grey rectangle). Bottom, RNA-seq (CPM) for embryos at 0-4 hpf and 4-8 hpf^147^; gene annotations. Positive-strand genes, orange; negative strand genes, blue. Dorsoventral patterning genes of interest, black. **a**, *Doc1*, *Doc2*, and *Doc3*. **b**, *dpp*. **c**, *sog*. **d**, *if*. **e**, *NetA* and *NetB*. **f**, *sna*.

Chromatin state and 3D organization are still being established at the cellular blastoderm stage^50,51,65,78,79^. Therefore, we carried out additional Hi-C experiments in control and *gd*^7^, *Toll*^rm9/rm10^ and *Toll*^10B^ embryos at a later developmental stage (stage 10, approximately 4–5 hpf) to assess whether tissue-specific genome organization develops later in development after the full establishment of histone modifications^65^. Aggregate analysis of compartments, domains and loops (Extended Data Fig. 8) revealed that these features were maintained across tissues at stage 10. Inspection of individual loci containing dorsoventral patterning genes (Extended Data Fig. 9) also confirmed that their chromatin organization was similar. A small number of loci showed tissue-specific changes, such as the *dpp* locus, at which a small domain encompassed the expressed portion of the gene in *gd*^7^ mutants but was absent in the other tissues. Overall, these results suggest that most developmentally regulated genes do not develop tissue-specific chromatin organization over this developmental period, further arguing that tissue-specific chromatin organization is not required for tissue-specific expression during this developmental transition.

### Micro-C reveals maintenance of fine scale chromatin conformation and enhancer–promoter interactions across tissues

**Fig. 5:**
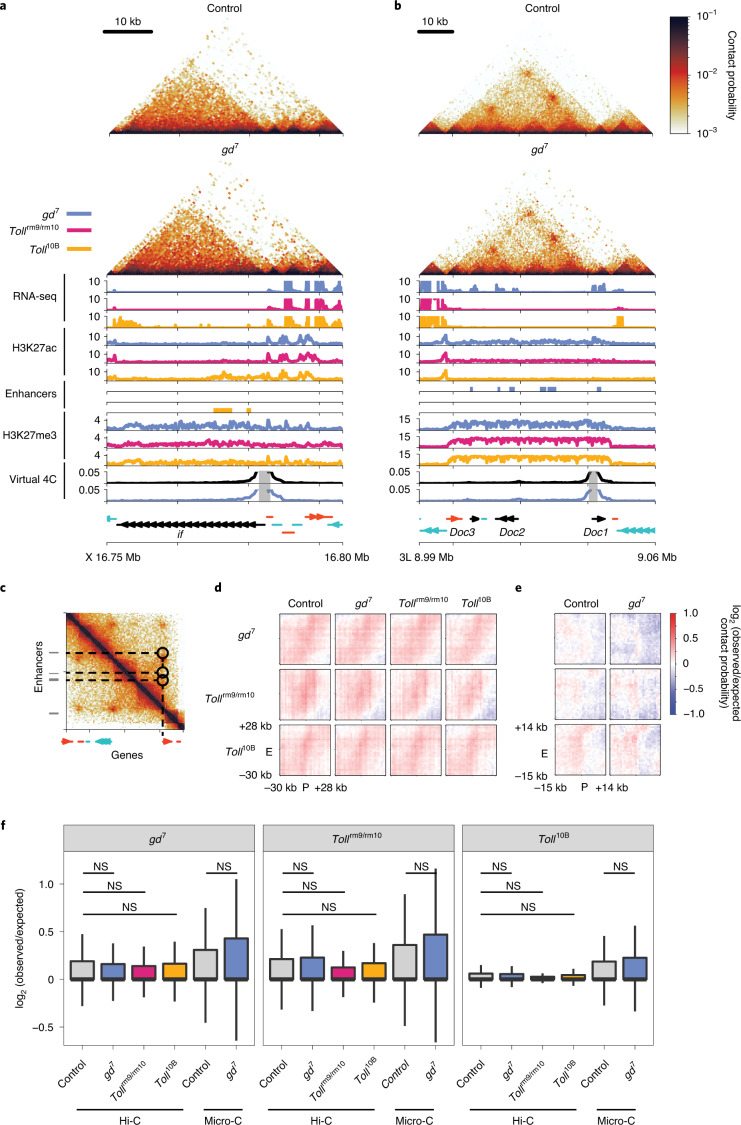
Enhancer–promoter interactions do not correlate with tissue-specific enhancer activity or gene expression. **a**,**b**, High-resolution chromatin organization at dorsoventral patterning genes (in black; *if*, **a**; *Doc1*, *Doc2*, *Doc3*, **b**). Top, normalized Micro-C contact probability at 500-bp resolution. RNA-seq (CPM) and H3K27ac and H3K27me3 ChIP-seq (CPM) data are shown in blue (*gd*^7^), pink (*Toll*^rm9/rm10^) and yellow (*Toll*^10B^) (ref. ^62^, this study). Tissue-specific putative enhancers are indicated by color-coded bars. ‘Virtual 4C’ tracks representing interactions of a 2-kb region around the promoter of *if* (**a**) or *Doc1* (**b**). Positive-strand genes, orange; negative-strand genes, blue. **c**, Schematic representation of construction of enhancer–promoter interaction aggregates shown in **d**,**e**. Enhancers (gray bars) were assigned to putative target genes (Methods). Enhancers within 5 kb of their assigned promoter were excluded. Regions of the interaction matrix corresponding to enhancer–promoter interactions (circles) were extracted and averaged across sets of tissue-specific enhancers. **d**,**e**, Aggregate contact analysis for putative tissue-specific enhancers (E) and the promoters (P) of their assigned genes. The average observed/expected contact probability is shown for Hi-C data at 2-kb resolution in a window of 60 kb around putative enhancer–promoter interactions (**d**) or for Micro-C data at 1-kb resolution in a window of 30 kb (**e**). Rows represent enhancer sets; columns represent genotypes. **f**, Quantification of contact probability between putative enhancers and their assigned target promoters. Panels represent enhancer sets; *x* axes represent Hi-C and Micro-C data from different genotypes. There were no significant differences in interaction strength between control and mutant datasets (two-sided Wilcoxon rank-sum test; *n* = 302 *gd*^7^ enhancer–gene pairs, *n* = 235 *Toll*^rm9/rm10^ enhancer–gene pairs, *n* = 337 *Toll*^10B^ enhancer–gene pairs). Box plots show median, box spanning first to third quartiles, whiskers extending to smallest or largest values no further than 1.5 × IQR from the box and notches extending 1.58 × IQR × (√*n*)^−1^ from the median. NS, not significant.

**Extended Data Fig. 10:**
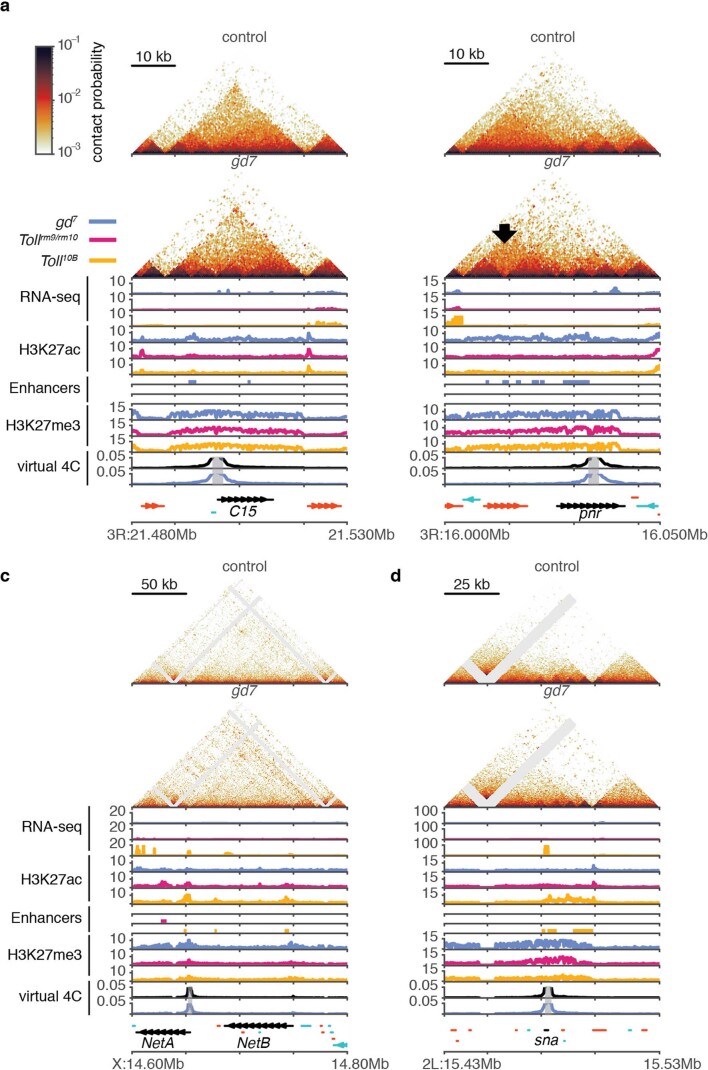
Micro-C confirms that high-resolution chromatin conformation is largely unaffected by tissue-specific gene expression and enhancer activity. Micro-C data for regions containing dorsoventral patterning genes. For control and *gd*^*7*^ embryos, top, normalized Micro-C contact probability, 500 bp resolution; middle, RNA-seq data (CPM)^62^; bottom, H3K27ac and H3K27me3 ChIP-seq data (CPM^62^, this study). Tissue-specific putative enhancers, color-coded bars beneath corresponding H3K27ac ChIP-seq track. Lower panels, ‘virtual 4C’ tracks representing interactions of a 2 kb region around the promoters of genes of interest (grey rectangle); gene annotations; positive-strand genes, orange; negative strand genes, blue. Dorsoventral patterning genes of interest, black. Arrow highlights a region in the *pnr* locus that gains insulation in *gd*^*7*^ embryos. **a**, *C15*. **b**, *pnr*. **c**, *NetA* and *NetB*. **d**, *sna*.

To obtain higher resolution data, we carried out whole-genome chromosome conformation capture using micrococcal nuclease digestion (Micro-C)^80^ in control and *gd*^7^ embryos at the cellular blastoderm stage. This allowed us to examine chromatin conformation at dorsoventral patterning genes at 500-bp resolution (Fig. 5a,b). At this resolution, additional structures were revealed. For example, the region adjacent to the *if* locus containing housekeeping genes appeared unstructured in Hi-C data, but several small domains (5–10 kb) were visible in Micro-C data (compare Fig. 1g and Fig. 5a). In addition, loops adjacent to the promoters of *Doc1*, *Doc2* and *Doc3* that were visible in Hi-C data from embryos at 3–4 hpf but not in cellular blastoderm embryos were apparent in Micro-C data from these embryos (compare Fig. 1h, Fig. 4e and Fig. 5b). However, some structures that were identified with Micro-C in mammalian systems were notably absent here; we did not detect ‘stripes’ originating from active promoters or prominent enhancer–promoter loops^20,21^. At this increased level of resolution, there were nevertheless few differences between control and *gd*^7^ chromatin organization at dorsoventral patterning genes (Fig. 5a,b and Extended Data Fig. 10), further supporting the idea that differential chromatin organization is not required for differential gene expression during dorsoventral patterning.

Finally, traditional models of gene regulation by enhancers predict that interactions between regulatory elements and their target promoters increase upon tissue-specific gene expression^81^. While punctate enhancer–promoter interactions were not visible in Hi-C or Micro-C data, and changes in enhancer–promoter interaction strength were not apparent upon visual examination (Figs. 4e and 5a,b and Extended Data Figs. 6 and 10), we performed aggregate analysis to systematically determine whether subtle changes in interaction strength would manifest at these loci (Fig. 5c). This revealed that there was no significant increase in Hi-C or Micro-C interaction frequency between enhancers and their target promoters in the tissue in which the enhancer was active (Fig. 5d–f). This suggests that increased enhancer–promoter interaction frequency was not required for the tissue-specific expression of dorsoventral patterning genes. In sum, our results provide evidence for the independence of tissue-specific gene expression and chromatin conformation during dorsoventral patterning.

## Discussion

Previous studies produced conflicting results regarding the relationship between gene expression, chromatin state and 3D chromatin organization. Here, we set out to understand this relationship in the context of embryonic development in *Drosophila*. Using the well-studied dorsoventral patterning system, we showed that, despite significant differences in chromatin state and gene expression between tissues along the dorsoventral axis of the embryo, chromatin conformation is largely maintained across tissues. This suggests that cell-type-specific gene regulation does not require cell-type-specific chromatin organization in this context. Nevertheless, developmentally regulated genes and enhancers are organized into chromatin domains. We suggest that this organization plays a permissive role to facilitate the precise regulation of developmental genes.

We made use of maternal effect mutations in the Toll signaling pathway, which lead to embryos that lack the usual patterning of the dorsoventral axis^53^ and have long been used as a system to study the specification of mesoderm (*Toll*^10B^), neuroectoderm (*Toll*^*r*m9/rm10^) and dorsal ectoderm (*gd*^7^) cell fates as well as the regulation of tissue-specific gene expression^60–64^. However, these embryos are still under the influence of anterior–posterior patterning signals and do not show completely uniform cell identities^60^. We sought to investigate heterogeneity of cell identity at the single-cell level by using single-cell gene expression profiling. This revealed that certain cell types are indeed maintained in all three Toll pathway mutants, including pole cells and other terminal region cell identities, hemocytes and trachea precursor cells (Fig. 2). However, heterogeneity of gene expression is reduced in the mutants, as shown by the loss of cells assigned to mesoderm clusters in *gd*^7^ and *Toll*^rm9/rm10^ embryos and the depletion of ectoderm subsets in each of the mutants. These datasets showcase the advantages of measuring cellular heterogeneity at the single-cell level and provide a useful resource for further characterization of these embryos and investigation of the regulation of dorsoventral patterning.

Although the *gd*^7^, *Toll*^rm9/rm10^ and *Toll*^10B^ embryos still have heterogeneous gene expression profiles, nevertheless, there are clear differences in chromatin state and overall gene expression between these embryos^60–63^. We expanded on previous studies by identifying putative enhancers specific to neuroectoderm in addition to dorsal ectoderm and mesoderm. This allowed the identification of tissue-specific putative enhancer–gene pairs, which correspond well with known dorsoventral patterning enhancers and genes that are DE across the dorsoventral axis. These regulatory elements and their target genes are located inside chromatin domains, distinct from the enrichment of housekeeping genes at domain boundaries^50,66,67,72,82,83^. This is in line with previous results that suggest that 3D chromatin domains act as regulatory domains^14,84–87^.

We find that this domain organization is maintained across tissues, even in cases in which there are significant changes in the local chromatin state and gene expression (Fig. 3 and Extended Data Fig. 4). This is consistent with earlier results from Hi-C experiments carried out in anterior and posterior embryo halves, which also showed no differences^88^, and with previous studies in *Drosophila* cell lines and other systems, which suggested that domains are widely conserved across different tissues and even different species^13,43,67,89^. To explain this maintenance of organization across cell lines, it was proposed that active chromatin, especially at broadly expressed genes, is responsible for partitioning the genome into domains^9,67^. Rowley et al.^9^ proposed that compartmentalization of active and inactive chromatin, at the level of individual genes, underlies the formation of insulated chromatin domains. This model predicts that, when a developmentally regulated gene is active, its domain would merge with or have increased interactions with neighboring domains containing active genes, such as broadly expressed housekeeping genes. Our results do not support this model, as we find no evidence that differences in domain structure are driven by changes in chromatin state or by active expression of developmentally regulated genes. By contrast, this supports the idea that, similar to mammalian domain architecture, additional factors, such as insulator proteins, modulate domain organization in *Drosophila*^2,90^. Therefore, based on current data, we do not believe that active transcription is the key determinant of 3D chromatin organization in this system.

While overall and locus-specific chromatin organization are maintained across tissues, our Hi-C and Micro-C analyses identify a small number of examples of regions that do have changes in organization (Extended Data Figs. 5, 8 and 9). However, at these loci, there is no clear relationship between changes in organization and changes in chromatin state or expression, and the vast majority of developmentally regulated loci in this system do not have changes. It will be important for future studies to further investigate these loci to understand what drives these rare changes.

We also investigated chromatin organization at the level of enhancer–promoter interactions. Previous studies produced conflicting results about whether these interactions are correlated with tissue-specific activation of gene expression. We found no evidence for widespread enrichment of interactions between enhancers and their target promoters, including in tissues where they are active. This is in contrast with previous studies using 3C approaches that have found evidence of enriched enhancer–promoter interactions^15,16,18,20,21^, which may precede^19,22^ or correlate with^40,45^ transcriptional activation. Notably, Ghavi-Helm et al.^19^ found that a subset of *Drosophila* long-range enhancer–promoter pairs do form stable interactions that are enriched above local background^19^. While these loops are visible in our dataset, our results suggest that such loops are not likely to be the primary mechanism of promoter regulation during *Drosophila* development, perhaps because most enhancers are close to their target promoters. Many stable loops in the *Drosophila* genome are instead associated with polycomb-mediated repression^51,91^.

Hi-C provides information about the average conformation across a population of hundreds of thousands of nuclei, which contain dynamic ensembles of different 3D conformations^90,92–100^. While our scRNA-seq results indicate that the mutant embryos contain a range of different cell types, we believe that our results indicate that the 3D chromatin structures in these cell types are drawn from the same population of possible conformations. This is supported by results from a recent study^101^ analyzing the structure of the *Doc* and *sna* loci in *Drosophila* embryos using Hi-M, a high-resolution single-cell imaging approach. Strikingly, this orthogonal technique also reveals chromatin organization that is consistent across different tissues in the embryo, despite differential expression of these genes. Imaging-based approaches directly measure spatial proximity between genomic loci, whereas Hi-C and Micro-C rely on cross-linking to detect chromatin interactions. Therefore, care must be taken when comparing these approaches. Nevertheless, both approaches indicate that genome organization is maintained across different tissues in this system.

Our results are consistent with several recent studies in mammals as well as in *Drosophila*, which provide evidence that stable enhancer–promoter contacts are not always required for gene activation^27–30,102^. This is in line with models in which transient or indirect contacts with a regulatory element are sufficient to activate transcription^102–106^, such as through the formation of nuclear microenvironments or phase-separated condensates^107–109^.

**Fig. 6:**
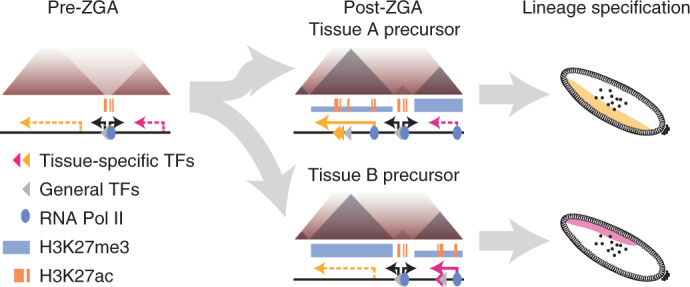
Model of the relationship between chromatin conformation and developmentally regulated gene expression. Left, before ZGA, the genome is unstructured, with domain boundaries appearing at a subset of regions associated with binding of RNA Pol II and Zelda. Middle, chromatin domains are established at ZGA, and domain structure is the same across tissues with different gene expression and transcription factor (TF) binding. Right, differential activity of regulatory elements in the context of the same chromatin conformation leads to different patterns of gene expression in the developing embryo. Thick and thin blue bars represent high and low levels of H3K27me3, respectively; dashed lines represent inactive genes, while solid lines represent actively transcribed genes.

Together, our results indicate that differential chromatin organization is not a necessary feature of cell-type-specific gene expression. We propose that chromatin organization into domains instead provides a scaffold or framework for the regulation of developmental genes during and after the activation of zygotic gene expression (Fig. 6, left and middle). This may help render developmental enhancers ‘poised’ for timely regulation of target genes upon receipt of appropriate cellular signals (Fig. 6, right). Other mechanisms of priming have been described, including paused polymerase (Pol) II at promoters^110,111^ and pioneer factors bound to poised enhancers^64,112,113^. Feedback effects, such as downstream modification of chromatin state and additional mechanisms, including looping between polycomb-bound elements and segregation of active and inactive chromatin, may then act as layers on top of the initially established domain structure.

## Methods

### *Drosophila* stock maintenance

yw; eGFP-PCNA flies used as controls for Hi-C and the first scRNA-seq control experiment were kindly provided by S.A. Blythe and E. Wieschaus (Princeton University)^78^ and maintained on standard cornmeal–agar food. The *w*^1118^ flies used for the second scRNA-seq control experiment and the *Toll* mutant fly stocks *gd*^7^/*winscy hs-hid, Toll*^10B^/*TM3 e Sb Ser*/*OR60* and *Toll*^rm9/rm10^/*TM6 e Tb Sb* were grown on potato mash–agar food. All fly stocks were incubated at 25 °C with a 12-hour light–dark cycle.

The embryos representing presumptive dorsal ectoderm were collected from *gd*^7^-homozygous flies. One-day-old larvae laid by *gd*^7^/*winscy hs-hid* flies were heat shocked for 1.5 h at 37 °C twice with a 24-h interval to eliminate *gd*^7^-heterozygous animals. Embryos from *Toll*^10B^/*TM3 e Sb Ser* or *Toll*^10B^/*OR60* heterozygous females represented presumptive mesoderm. *Toll*^rm9^/*Toll*^rm10^ trans-heterozygous females were used for collecting presumptive neuroectoderm embryos.

### Chromatin immunoprecipitation sequencing

*Toll*^rm9^/*Toll*^rm10^ 2-4-h-old embryos were collected for ChIP-seq and fixed as described above for Hi-C. Fixed embryos were flash frozen in liquid nitrogen and stored at −80 °C until further use. Frozen embryos were homogenized in sonication buffer (50 mM HEPES, 140 mM NaCl, 1 mM EDTA, 1% Triton, 0.1% sodium deoxycholate, 0.1% SDS and protease inhibitor) using a Dounce homogenizer. The samples were centrifuged at 4,000*g* for 5 min, and the pellets containing the intact nuclei were resuspended in the same buffer supplemented with 0.5% *N*-lauroylsarcosine and SDS to a final concentration of 0.5%. Chromatin was sheared to a fragment size in the range of 200–500 bp using a Bioruptor (Diagenode). The solubilized chromatin fraction was cleared by centrifugation and used for immunoprecipitation after diluting it five times with sonication buffer. Immunoprecipitation with either 2 µg anti-H3K27ac (Abcam, ab4729) or 5 µg anti-H3K27me3 (Abcam, ab6002) antibody was carried out on chromatin corresponding to 20–25 µl of embryos at 4 °C overnight. Chromatin–antibody complexes were captured for at least 3 h using a mix of Protein A and G Dynabeads (Invitrogen). The captured immunoprecipitated complexes were washed 10 min with each of the following: sonication buffer (50 mM HEPES, 140 mM NaCl, 1 mM EDTA, 1% Triton, 0.1% sodium deoxycholate, 0.1% SDS), ‘WashA’ (same composition as for sonication buffer but with 500 mM NaCl), ‘WashB’ (20 mM Tris, pH 8, 1 mM EDTA, 250 mM LiCl, 0.5% NP-40, 0.5% sodium deoxycholate) and TE. After the washes, Dynabeads with bound chromatin–antibody complexes were resuspended in 100 µl TE supplemented with 20 mg ml^−1^ RNase A and incubated at 50 °C for 30 min. Cross-linking was reversed by adding Tris, pH 8.0 and SDS to a final concentration of 50 mM and 0.1%, respectively, and heating at 68 °C for at least 4 h. Protein digestion was carried out by treatment with proteinase K at 55 °C for 2 h, followed by purifying chromatin-immunoprecipitated DNA using the ChIP DNA Clean & Concentrator kit (Zymo Research, D5205). ChIP-seq libraries were prepared on the chromatin-immunoprecipitated DNA eluted in 60 µl DNA elution buffer, using the NEBNext Ultra II DNA Library Prep kit (NEB). ChIP samples were single-end (1 × 75 bp) sequenced on the Illumina NextSeq platform at the BEA core facility, Stockholm.

### Single-cell RNA sequencing

We adapted the collection and methanol fixation procedures described in refs. ^71,119^. Following a precollection period of at least 1 h, fly embryos were collected on yeasted apple juice plates at 25 °C. After 1 h of collection, the embryos on the plate were incubated at 25 °C for 2.25 h. Embryos were dechorionated for 2 min in 2.6% sodium hypochlorite, rinsed with water and suspended in PBS, 0.5% Triton X-100. Embryos were rinsed with cell culture-grade DPBS without Ca^2+^ and Mg^2+^ to remove residual detergent and placed on ice at precisely 3.5 h after the start of collection. Embryos were resuspended in 500 µl ice-cold dissociation buffer (cell culture-grade DPBS without Ca^2+^ and Mg^2+^, 0.04% BSA) and dissociated with a clean metal pestle. Cells and tissue fragments were pelleted at 500*g* for 5 min at 4 °C and then gently resuspended in 100 µl trypsin-EDTA (0.25%) and incubated for 3 min. After 3 min, trypsin was quenched by adding 1 ml cell culture-grade DPBS (without Ca^2+^ and Mg^2+^), 10% FCS. Cells were pelleted at 1,000*g* for 5 min at 4 °C and then resuspended in 500 µl dissociation buffer, pelleted again and resuspended in 100 µl dissociation buffer. A 10-µl aliquot of cells was kept and counted using an improved Neubauer chamber or a Luna2 cell counter. To fix cells, four volumes of 100% methanol, prechilled at −20 °C, were slowly added to the cells. Fixed cells were stored at −80 °C and used within 3 d.

scRNA-seq was performed using the 10x Genomics Chromium Single Cell 3′ Reagents version 3, according to the manufacturer’s instructions (revision B). Methanol-fixed cells were centrifuged at 3,000*g* and 4 °C for 5 min and resuspended in 500 µl DPBS with 0.04% BSA to rehydrate. Rehydrated cells were counted using a Luna2 cell counter, and the volume used for library preparation was chosen for a targeted recovery of 5,000 cells. Libraries were sequenced on an Illumina NextSeq 500, using paired-end sequencing (read 1 length, 28 cycles; index read length, eight cycles; read 2 length, 91 cycles).

### Hi-C

We adapted the fixation and sorting procedure described in ref. ^117^ for in situ Hi-C^120,121^. Following a precollection period of at least 1 h, fly embryos were collected on yeasted 0.4% acetic acid agar plates or apple juice plates at 25 °C. After 1 h of collection, the embryos on the plate were incubated at 25 °C for 2 h for collection of cellular blastoderm embryos and 4 h for collection of stage 10 embryos. Embryos were dechorionated for 2 min in 2.6% sodium hypochlorite, rinsed with water and transferred to vials containing 2 ml PBS, 0.5% Triton X-100 and 6 ml heptane. Cross-linking was initiated by adding 100 µl 37% formaldehyde, followed by vigorous shaking. After 10 min, samples were centrifuged at 500*g* for 1 min, and the upper heptane layer was removed. Fifteen minutes after the start of fixation, 5 ml PBS, 0.5% Triton X-100, 125 mM glycine was added to the embryos, followed by vigorous shaking for 1 min. The embryos were rinsed three times with PBS, 0.5% Triton X-100. Embryos were sorted in small batches under a light microscope, based on morphology, to select embryos of the appropriate developmental stage and remove damaged embryos or embryos with abnormal morphology. Embryos from *gd*^7^, *Toll*^rm9/rm10^ and *Toll*^10B^ mutant mothers do not gastrulate correctly and do not have normal morphology at stage 10; therefore, sorting of these embryos largely consisted of removing embryos that were clearly dead, dying or from earlier or later stages. Sorted embryos were aliquoted such that a single tube contained enough embryos for one experiment and then were flash frozen in liquid nitrogen and stored at −80 °C. We used 30–60 embryos for each in situ Hi-C experiment.

In situ Hi-C was performed according to the protocol in refs. ^50,120,121^, using MboI as the restriction enzyme, with minor modifications to optimize for low input according to ref. ^122^.

### Micro-C

Embryos were collected as described above for Hi-C with minor modifications. Following the first cross-linking with formaldehyde, the reaction was quenched with 2 M Tris-HCl, pH 7.5 (final concentration, 0.75 M). Embryos were washed with PBST, and a second cross-linking step was carried out using long cross-linkers DSG and EGS (Sigma) at a final concentration of 3 mM in PBST for 45 min at room temperature with passive mixing. The reaction was quenched again with Tris-HCl, pH 7.5 at a final concentration of 0.75 M for 5 min. Embryos were washed, sorted under a microscope, snap frozen with liquid nitrogen and stored at −80 °C. Micro-C libraries were constructed according to ref. ^21^ with modifications. At least 300 nc14 embryos were used per library. Embryos were crushed in the Eppendorf tube with liquid nitrogen-cooled plastic pestles using 500 µl buffer MB1 (50 mM NaCl, 10 mM Tris, 5 mM MgCl_2_, 1 mM CaCl_2_, 0.2% NP-40, 1× PIC). Chromatin was digested with a predetermined amount of Micrococcal Nuclease (Worthington Biochemical) to yield 90% monomer versus 10% dimer, given the appropriate number of embryos. When embryos were limited, as in *gd*^7^ libraries, size selection of dinucleosomal DNA was not carried out by gel extraction. Instead, total DNA was carried over into the final library construction phase and finally size selected for the appropriate dinucleosomal band (350–500 bp) on a 3.5% NuSieve agarose gel after PCR. Libraries were paired-end sequenced on an Illumina NovaSeq S1 100 nt Flowcell (read 1 length, 50 cycles; index read length, six cycles; read 2 length, 50 cycles).

### ChIP-seq analysis

ChIP-seq reads were mapped to the dm6 genome using Bowtie 2, version 2.3.3.1 (ref. ^123^). Mapped reads were filtered to remove alignments with quality scores less than 30, as well as secondary and supplementary alignments. PCR duplicates were marked using sambamba version 0.6.8 (ref. ^124^). Coverage tracks were generated using the bamCoverage tool from deepTools version 3.2.0 (ref. ^116^) with the following parameters: ‘-of bigwig --binSize 10 --normalizeUsing CPM --extendReads 200 --ignoreDuplicates --minMappingQuality 30’, and reads were only kept from chromosomes X, 2L, 2R, 3L, 4 and Y. ChIP-seq peaks were called using MACS2 version 2.2.6 (ref. ^125^) with the following parameters: ‘--nomodel --extsize 147 -g dm’ or ‘--nomodel --extsize 147 -g dm --broad --min-length 500 --max-gap 200’ for broad peaks. We used merged input samples for each genotype as the controls for all peak calling due to a lack of sample-matching information for the published datasets that were reanalyzed.

### RNA-seq analysis

RNA-seq reads were quantified using Salmon 1.1.0 (ref. ^126^) and the Flybase r6.30 transcripts. Salmon was used in mapping-based mode, with the following parameters: ‘-l A --validateMappings –seqBias’. For visualization purposes, RNA-seq reads were also aligned to the dm6 genome, using HISAT2 version 2.1.0 (ref. ^127^). Mapped reads were filtered to remove alignments with quality scores less than 30, as well as secondary and supplementary alignments. PCR duplicates were marked using sambamba version 0.6.8 (ref. ^124^). Coverage tracks were generated using the bamCoverage tool from deepTools version 3.2.0 (ref. ^116^) with the following parameters: ‘-of bigwig --binSize 10 --normalizeUsing CPM --extendReads 200 --ignoreDuplicates --minMappingQuality 30’, and reads were only kept from chromosomes X, 2L, 2R, 3L, 4 and Y.

We used tximport version 1.14.2 (ref. ^128^) to import quantifications from Salmon into R (3.6.3) and estimate transcripts per million values. Pairwise differential expression analysis was carried out between *gd*^7^, *Toll*^rm9/rm10^ and *Toll*^10B^ embryos using DESeq2 version 1.26.0 (ref. ^129^) with default parameters.

### Identification of candidate tissue-specific enhancers

To identify tissue-specific enhancers, pairwise differential H3K27ac signal analysis was first carried out using csaw version 1.20.0 (refs. ^114,115^) and edgeR version 3.28.1 (ref. ^130^). We used 2,000-bp windows for the background calculations and selected 150-bp windows with a 2.5-fold enrichment over the background. Windows were merged using the parameters ‘tol = 100’ and ‘max.width = 5000’. Merged regions with a false discovery rate <0.05 and with a consistent direction of change across all windows were selected for downstream analysis. Candidate tissue-specific enhancers were defined by taking the intersection of regions identified as enriched for H3K27ac in each genotype compared to both of the other genotypes.

We validated the putative enhancers by comparing them to enhancers identified in previous studies. Six of 22 dorsal ectoderm enhancers identified from a literature search^62^ overlapped with our *gd*^7^-specific enhancers, while ten of 37 mesoderm enhancers overlapped with our *Toll*^10B^-specific enhancers (Extended Data Fig. 1c). The relatively low overlap can be explained by the fact that many literature enhancers have H3K27ac signal in *Toll*^rm9/rm10^ mutants as well as in either *gd*^7^ or *Toll*^10B^ mutants. Putative enhancers were also overlapped with regions tested for enhancer activity in *Drosophila* embryos in ref. ^131^. Regions (‘tiles’) tested by Kvon et al. that were active in at least one tissue and time point were lifted over to dm6 from dm3. One hundred and sixty-five putative enhancers overlapped a total of 183 tiles by at least 1 bp. Of 27 *gd*^7^ enhancers, which overlap tiles that are active in stages 4–6 or stages 7–8, 19 were active in either dorsal ectoderm or amnioserosa precursors and/or subsets. Of 35 *Toll*^rm9/rm10^ enhancers, which overlap tiles that are active in stages 4–6 or stages 7–8, 26 were active in brain or ventral nerve cord precursors, procephalic ectoderm or ventral ectoderm. Of the 35 *Toll*^10B^ enhancers, which overlap tiles that are active in stages 4–6 or stages 7–8, 27 were active in mesoderm precursors and/or subsets.

Enhancer heatmaps were made using the plotHeatmap tool from deepTools version 3.2.0 (ref. ^116^). Overlaps between different enhancer sets were visualized using UpSetR version 1.4.0 (refs. ^132,133^).

### Assignment of candidate enhancers to target genes

We defined ‘housekeeping genes’ as genes that have at least ‘low’ expression in all stages and tissues according to Flybase RNA-seq data (1,867 genes). We filtered the set of genes from the Flybase 6.30 transcripts to remove these housekeeping genes, as well as any genes with an average transcripts per million value <1 in the *gd*^7^, *Toll*^rm9/rm10^ and *Toll*^10B^ bulk RNA-seq data. Candidate tissue-specific enhancers were assigned to target genes using the following rules: first, we assigned any enhancers that overlapped a single transcript to that gene. Next, we assigned enhancers to the closest promoter that was not separated from the enhancer by a domain boundary (using consensus boundaries from embryos at 3–4 hpf, see below). The remaining enhancers were assigned to the closest promoter within the same domain or, if they were not inside a domain, to the closest promoter.

### scRNA-seq analysis

We used Cell Ranger (version 3.1.0) to produce FastQ files for the scRNA-seq data and to align, filter and quantify reads based on the BDGP6.22 genome release (Ensembl 98) to produce feature–barcode matrices. We imported the filtered matrices into R using DropletUtils version 1.6.1 (ref. ^134^) and performed additional quality control analysis using scater (version 1.14.6 (ref. ^135^)). Doublets were identified using scDblFinder version 1.1.8 (ref. ^136^), with an estimated doublet rate of 3.9%, and removed. Normalization for library size across cells was performed using scater and scran version 1.14.6 (ref. ^137^) using the ‘deconvolution’ approach described in ref. ^138^, in which cells are preclustered and size factors are estimated using the calculateSumFactors() function.

Downstream analysis was carried out using Seurat version 3.1.4 (refs. ^139,140^). The VST method was used to select the top 3,000 variable features for each sample, and then all datasets were integrated using the control dataset with the highest number of cells (replicate 1) as the reference dataset and the first 30 dimensions. We performed clustering using the shared nearest neighbor approach implemented in the Seurat functions FindNeighbors and FindClusters, using the first 12 dimensions from PCA, *a k* parameter value of 60 and a clustering resolution of 0.5. These parameters were chosen because they produced clusters that were stable to small variations in the parameter values.

Differential expression analysis was carried out using the Seurat function FindMarkers to identify genes with higher expression in a cluster compared to those in all other cells and in pairwise comparisons. We carried out Gene Ontology enrichment analysis on the resulting marker gene sets using the enrichGO function from clusterProfiler version 3.14.3 (ref. ^141^) and simplified the results to remove semantically similar terms using the ‘simplify’ function from clusterProfiler with the Wang method and a similarity threshold of 0.7. These marker gene sets and enriched GO terms, along with the expression of known markers for embryonic cell populations, were used to assign putative cluster identities.

To quantify the average expression of particular gene sets in Fig. 2c and Extended Data Fig. 3c, we calculated the sum of the expression of these genes per cell and then expressed this as a *Z* score across all cells.

Pooled scRNA-seq reads from all barcodes were analyzed using Salmon as described above.

### Hi-C analysis

Hi-C data were analyzed using FAN-C version 0.8.28 (ref. ^142^). Paired-end reads were scanned to identify ligation junctions, split at ligation junctions, if any were present, and then aligned independently to the dm6 genome using BWA-MEM (version 0.7.17-r1188)^143^. Aligned reads were filtered to retain only uniquely aligned reads with a mapping quality of at least three. Reads were then paired based on read names and assigned to restriction fragments. ‘Inward’ and ‘outward’ reads separated by less than 1 kb, representing likely unligated fragments and self-ligated fragments, respectively, were removed. In addition, we removed PCR duplicates, reads mapping more than 500 bp from a restriction site and self-ligations, for which both reads map to the same fragment.

We generated two biological replicate datasets for each genotype, which showed high similarity. Therefore, we pooled biological replicates to reach 2-kb resolution. Matrices created from merged biological replicates were binned and filtered using FAN-C default parameters to remove bins with coverage less than 10% of the median coverage. Normalization was performed using Knight–Ruiz matrix balancing^144^. Expected contacts were calculated as the average contacts at each genomic distance separation. Hi-C data were visualized using plotting tools from FAN-C and using HiGlass^145^.

### Micro-C analysis

Micro-C analysis was performed using FAN-C in the same way as for the Hi-C analysis, except that reads were assigned to 100-bp genomic bins rather than restriction enzyme fragments, and ‘inward’ and ‘outward’ reads assigned to the same or adjacent bins (separated by less than 50 bp) were removed. Normalization was performed using iterative correction^146^, as Knight–Ruiz matrix balancing had prohibitive memory requirements at high resolution.

### Domain and boundary identification

The insulation score was calculated as described in ref. ^77^, using FAN-C, for 2-kb and 5-kb resolution matrices, each with window sizes of four, six, eight and ten bins. Domain boundaries were calculated from the insulation score with a delta parameter of three and filtered to keep only boundaries with a boundary score of at least 0.7. Consensus boundaries for each sample were created by overlapping boundaries called at the two different resolutions and four different window sizes and keeping the boundaries that were identified using at least four of the total eight parameter combinations. Domains were created by pairing boundaries, and domains less than 10 kb or more than 500 kb in size were removed.

### Hi-C aggregate analysis

Aggregate compartment, domain and loop plots were created using FAN-C. Compartment analysis was carried out using Hi-C matrices that had been masked to remove pericentromeric heterochromatin. Pericentromeric heterochromatin was identified using H3K9me3 ChIP-seq data from embryos at 0–4 hpf and 4–8 hpf from modENCODE^147^. H3K9me3 ChIP-seq data were processed as described above and binned at 10-kb resolution, and bins with enrichment for H3K9me3 compared to input in embryos at both 0–4 hpf and 4–8 hpf were selected. Bins closer than 25 kb were merged, regions smaller than 20 kb were removed, and remaining large regions within 100 kb were merged. This produced a small number of regions, of which one per chromosome clearly corresponded to the pericentromeric heterochromatin. Compartments were identified using the first eigenvector of the correlation matrix of the normalized Hi-C data, using GC content to orient the eigenvector. The compartment eigenvector for the 3–4 hpf Hi-C data from Hug et al.^50^ was used as the reference for the aggregate compartment plots (‘saddle plots’)^74,98,146,142^. Compartment aggregates were plotted with all regions with an eigenvector value of zero collapsed so that they represented a single row or column in the aggregate matrix. Domain aggregates were also created using the domains identified in the Hi-C data from Hug et al. for embryos at 3–4 hpf. Loop aggregates were created using the loops identified in Kc167 cells in ref. ^72^. Similar results were obtained using loops from refs. ^88,91^ (not shown).

We constructed a BEDPE file of putative enhancer–promoter interactions, considering all unique transcription start sites for assigned target genes. Interactions with a separation of at least 10 kb were used to create enhancer–promoter aggregate plots.

### Hi-C similarity score analysis with CHESS

We used CHESS version 0.2.0 (ref. ^75^) to compare Hi-C data from embryos of different genotypes. Briefly, CHESS treats Hi-C interaction matrices as images and applies the concept of the ssim, which is widely used in image analysis. We applied CHESS to 5-kb resolution Hi-C matrices using windows of 500 kb and a step size of 5 kb to produce similarity scores for pairwise Hi-C comparisons. Hi-C data from stage 5 control embryos were used as the reference dataset and compared to data from stage 5 dorsoventral mutant embryos and nc14 control embryos from Hug et al.^50^. To identify regions of the genome with significant changes between the reference and query datasets, we selected regions with an ssim *Z* score less than −2 and a signal-to-noise ratio *Z* score of at least 1. For the box plots in Fig. 4c, windows were classified as containing DE genes if they contained at least one gene that was significantly upregulated or downregulated in the query genotype compared to both of the other genotypes and had no DE genes otherwise. Only every hundredth window was considered, in order to use only non-overlapping windows. The final numbers of windows considered are as follows: *gd*^7^, 101 DE genes, 124 non-DE genes; *Toll*^rm9/rm10^, 74 DE genes, 152 non-DE genes; *Toll*^10B^, 183 DE genes, 41 non-DE genes. CHESS is available at https://github.com/vaquerizaslab/chess.

### Statistics and visualization

Statistical tests were carried out in R (version 3.6.3), and visualization was performed using the ggplot2 package^148^. Box plots are defined with boxes spanning the first to third quartiles. The whiskers extend from the box to the smallest or largest values no further than 1.5 × IQR away from the box. The notches extend 1.58 × IQR × (√*n*)^−1^ from the median. All statistical tests are two-sided.

### Reporting Summary

Further information on research design is available in the Nature Research Reporting Summary linked to this article.

## Online content

Any methods, additional references, Nature Research reporting summaries, source data, extended data, supplementary information, acknowledgements, peer review information; details of author contributions and competing interests; and statements of data and code availability are available at 10.1038/s41588-021-00799-x.

## Supplementary information

Reporting Summary

Peer Review Information

Supplementary Tables Supplementary Tables 1–4

## Data Availability

The Hi-C, Micro-C, scRNA-seq and ChIP-seq data produced in this study were submitted to ArrayExpress and are available under the following accession numbers: E-MTAB-9306, E-MTAB-9784, E-MTAB-9304 and E-MTAB-9303, respectively. In addition, we analyzed data from the following publicly available datasets: GEO accessions GSE68983, GSE18068 and GSE16013 and ArrayExpress accession E-MTAB-4918. Datasets are listed in full in Supplementary Table 4. Genome sequences and gene annotations were obtained from Flybase r6.30 (http://www.flybase.org) and Ensembl version 98 (http://www.ensembl.org/).

## Code availability

All computational analysis is described in the Methods, and code is also available at https://github.com/vaquerizaslab/Ing-Simmons_et_al_dorsoventral_3D_genome (archived on Zenodo at 10.5281/zenodo.4272001).
