# Supplementary material for: Independence of chromatin conformation and gene regulation during Drosophila dorsoventral patterning
Source: Nat Genet. 2021 Apr 1;53(4):487–99. doi: 10.1038/s41588-021-00799-x (PMC8035076; doi:10.1038/s41588-021-00799-x)
Supplement: Supplementary file 1 — Reporting Summary [file 41588_2021_799_MOESM1_ESM.pdf]

nature research

Corresponding author(s): Vaquerizas

Last updated by author(s): Feb 16, 2021

Reporting Summary

Nature Research wishes to improve the reproducibility of the work that we publish. This form provides structure for consistency and transparency in reporting. For further information on Nature Research policies, see our Editorial Policies and the Editorial Policy Checklist.

Statistics

For all statistical analyses, confirm that the following items are present in the figure legend, table legend, main text, or Methods section.

n/a	Confirmed	
☐	☒	The exact sample size (*n*) for each experimental group/condition, given as a discrete number and unit of measurement
☒	☐	A statement on whether measurements were taken from distinct samples or whether the same sample was measured repeatedly
☐	☒	The statistical test(s) used AND whether they are one- or two-sided<br>*Only common tests should be described solely by name; describe more complex techniques in the Methods section.*
☒	☐	A description of all covariates tested
☒	☐	A description of any assumptions or corrections, such as tests of normality and adjustment for multiple comparisons
☐	☒	A full description of the statistical parameters including central tendency (e.g. means) or other basic estimates (e.g. regression coefficient) AND variation (e.g. standard deviation) or associated estimates of uncertainty (e.g. confidence intervals)
☐	☒	For null hypothesis testing, the test statistic (e.g. F, t, r) with confidence intervals, effect sizes, degrees of freedom and P value noted<br>*Give P values as exact values whenever suitable.*
☒	☐	For Bayesian analysis, information on the choice of priors and Markov chain Monte Carlo settings
☒	☐	For hierarchical and complex designs, identification of the appropriate level for tests and full reporting of outcomes
☒	☐	Estimates of effect sizes (e.g. Cohen's *d*, Pearson's *r*), indicating how they were calculated

Our web collection on statistics for biologists contains articles on many of the points above.

Software and code

Policy information about availability of computer code

Data collection

No software was used for data collection.

Data analysis

All computational analysis is described in the Methods, and code is also available at github.com/vaquerizaslab/Ing-Simmons_et_al_dorsoventral_3D_genome (archived on Zenodo, DOI: 10.5281/zenodo.4272002).

Bowtie2 version 2.3.3.1, Langmead and Salzberg, 2012.
sambamba version 0.6.8, Tarasov et al., 2015.
deepTools version 3.2.0, Ramírez et al., 2014
MACS2 version 2.2.6, Feng et al., 2012
Salmon 1.1.0, Patro et al., 2017
Hisat2 version 2.1.0, Kim et al., 2019.
FAN-C version 0.8.28 Kruse et al., 2020.
BWA-MEM version 0.7.17-r1188 Li and Durbin, 2009.
CHESS version 0.2.0, Galan, Machnik et al., 2020.

R 3.6.3
tximport version 1.14.2, Soneson et al., 2016
DESeq2 version 1.26.0, Love et al., 2014
csaw version 1.20.0, Lun and Smyth, 2014; Lun and Smyth, 2016
edgeR version 3.28.1, Robinson et al., 2010.
UpSetR version 1.4.0, Conway et al., 2017; Lex et al., 2014.
CellRanger version 3.1.0
DropletUtils version 1.6.1, Griffiths et al., 2018,

scater version 1.14.6, McCarthy et al., 2017.
scDblFinder version 1.1.8, Germain et al., 2020,
scran version 1.14.6, Lun et al., 2016
Seurat version 3.1.4, Butler et al., 2018; Stuart et al., 2019.
clusterProfiler version 3.14.3, Yu et al., 2012
ggplot2 version 3.3.2, Wickham 2016

For manuscripts utilizing custom algorithms or software that are central to the research but not yet described in published literature, software must be made available to editors and reviewers. We strongly encourage code deposition in a community repository (e.g. GitHub). See the Nature Research guidelines for submitting code & software for further information.

Data

Policy information about availability of data

All manuscripts must include a data availability statement. This statement should provide the following information, where applicable:

- Accession codes, unique identifiers, or web links for publicly available datasets
- A list of figures that have associated raw data
- A description of any restrictions on data availability

The Hi-C, Micro-C, scRNA-seq, and ChIP-seq data produced in this study have been submitted to ArrayExpress and are available with the following accession numbers: E-MTAB-9306, E-MTAB-9784, E-MTAB-9304, and E-MTAB-9303 respectively. In addition, we analysed data from the following publicly available datasets: GEO accessions GSE68983, GSE18068, and GSE16013 and ArrayExpress accession E-MTAB-4918. Datasets are listed in full in Table S4. Genome sequences and gene annotations were obtained from Flybase r6.30 (flybase.org) and Ensembl version 98 (www.ensembl.org).

Field-specific reporting

Please select the one below that is the best fit for your research. If you are not sure, read the appropriate sections before making your selection.

☒ Life sciences ☐ Behavioural & social sciences ☐ Ecological, evolutionary & environmental sciences

For a reference copy of the document with all sections, see nature.com/documents/nr-reporting-summary-flat.pdf

Life sciences study design

All studies must disclose on these points even when the disclosure is negative.

Sample size	No statistical methods were used to predetermine sample size. Sample sizes were determined based on the general variability of these data types in the literature and are sufficient to reach conclusions.
Data exclusions	No data were excluded from the analyses.
Replication	Principal component analysis and correlations were used to assess similarity of biological replicates and confirmed that replication was successful. At least two replicate experiments using independent embryo batches were carried out for ChIP-seq, Hi-C, and Micro-C. Four replicates were obtained for Micro-C in control embryos. Only one replicate was carried out for scRNA-seq of mutant embryos due to the difficulties in obtaining sufficient input material. Analyses of dorsoventral enhancers were confirmed using an independent set of putative enhancers identified by Koenecke et al. 2016.
Randomization	Samples were assigned to experimental groups based on genotype; each sample included multiple mixed-sex embryos of the same developmental stage.
Blinding	The authors were not blinded to allocation during experiments and analysis as knowledge of group labels was necessary to carry out the research and experiments and analysis were standardised across all samples.

Reporting for specific materials, systems and methods

We require information from authors about some types of materials, experimental systems and methods used in many studies. Here, indicate whether each material, system or method listed is relevant to your study. If you are not sure if a list item applies to your research, read the appropriate section before selecting a response.

Materials & experimental systems

n/a	Involved in the study
☐	☒ Antibodies
☒	☐ Eukaryotic cell lines
☒	☐ Palaeontology and archaeology
☐	☒ Animals and other organisms
☒	☐ Human research participants
☒	☐ Clinical data
☒	☐ Dual use research of concern

Methods

n/a	Involved in the study
☐	☒ ChIP-seq
☒	☐ Flow cytometry
☒	☐ MRI-based neuroimaging

Antibodies

Antibodies used: H3K27ac (Abcam, ab4729, Lot No: GR312658); H3K27me3 (Abcam, ab6002, Lot No: GR275911)

Validation: Antibodies were validated by manufacturer for ChIP using mouse, cow, and human samples, and are predicted to react with Drosophila samples. No further validation was carried out.

Animals and other organisms

Policy information about studies involving animals; ARRIVE guidelines recommended for reporting animal research

Laboratory animals: The following Drosophila melanogaster strains were used:
yw; eGFP-PCNA
gd7/winscy hs-hid
Toll10B/TM3 e Sb Ser/OR60
Tollrm9/rm10/TM6 e Tb Sb
w1118
All samples were mixed-sex embryos. Depending on the experiment, the embryos were approximately 2-4 hpf or 4-5 hpf.

Wild animals: No wild animals were used in this study.

Field-collected samples: This study did not involve samples collected in the field.

Ethics oversight: Drosophila melanogaster culture was performed in accordance with local and national requirements. No specific ethical approval is required for experiments with Drosophila embryos.

Note that full information on the approval of the study protocol must also be provided in the manuscript.

ChIP-seq

Data deposition

☒ Confirm that both raw and final processed data have been deposited in a public database such as GEO.

☒ Confirm that you have deposited or provided access to graph files (e.g. BED files) for the called peaks.

Data access links
May remain private before publication.: ChIP-seq data is available in ArrayExpress accession E-MTAB-9303. Enhancer calls are available in Supplementary Table 1. Peak calls were used only for quality control and not for supporting results.

Files in database submission: fastq

Genome browser session (e.g. UCSC): We do not have a genome browser session with this data.

Methodology

Replicates: Two biological replicates were produced.

Sequencing depth: All ChIP-seq samples were single-end sequenced to 75bp. The numbers of reads and percentage aligning uniquely are as follows: H3K27ac Rep1: 17 million, 80%; H3K27ac Rep2: 39 million, 76%; H3K27me3 Rep1: 35 million, 72 %; H3K27me3 Rep2: 31 million, 36%.

Antibodies: H3K27ac (Abcam, ab4729, Lot No: GR312658); H3K27me3 (Abcam, ab6002, Lot No: GR275911)

Peak calling parameters: ChIP-seq reads were mapped to the dm6 genome using Bowtie2 (version 2.3.3.1 (Langmead and Salzberg, 2012)). Mapped reads were filtered to remove alignments with quality scores less than 30, as well as secondary and supplementary alignments. PCR duplicates were marked using sambamba (version 0.6.8, (Tarasov et al., 2015)). Coverage tracks were generated using the bamCoverage tool from deepTools (version 3.2.0, (Ramírez et al., 2014)) with the following parameters: "-of bigwig -- binSize 10 -- normalizeUsing CPM --extendReads 200 --ignoreDuplicates --minMappingQuality 30" and keeping only reads from chromosomes X,

2L, 2R, 3L, 4, and Y. ChIP-seq peaks were called using MACS2 (version 2.2.6, (Feng et al., 2012)) with the following parameters: "--nomodel --extsize 147 -g dm " or "--nomodel --extsize 147 -g dm --broad --min-length 500 --max-gap 200" for broad peaks. We used merged input samples for each genotype as the controls for all peak calling, due to a lack of sample- matching information for the published datasets that were re-analysed.

Data quality

Aligned reads with alignment quality less than 30 were excluded. H3K27ac ChIP-seq had 4607 (Rep1) and 3255 (Rep2) peaks with FDR <=0.05 and fold enrichment >= 5; H3K27me3 ChIP-seq had 423 (Rep1) and 1325 (Rep2) peaks with FDR <=0.05 and fold enrichment >= 5.

Software

Bowtie2 version 2.3.3.1, Langmead and Salzberg, 2012.
sambamba version 0.6.8, Tarasov et al., 2015.
deepTools version 3.2.0, Ramírez et al., 2014
MACS2 version 2.2.6, Feng et al., 2012
