# Supplementary material for: Independence of chromatin conformation and gene regulation during Drosophila dorsoventral patterning
Source: Nat Genet. 2021 Apr 1;53(4):487–99. doi: 10.1038/s41588-021-00799-x (PMC8035076; doi:10.1038/s41588-021-00799-x)
Supplement: Supplementary file 2 — Peer Review Information [file 41588_2021_799_MOESM2_ESM.pdf]

Peer Review Information

Journal: Nature Genetics
Manuscript Title: Independence of chromatin conformation and gene regulation during *Drosophila* dorsoventral patterning
Corresponding author name(s): Dr. Juan Vaquerizas

Editorial Notes:

N/A

Reviewer Comments & Decisions:

Decision Letter, initial version:

13th Aug 2020

Dear Juanma,

Your Article, "Independence of 3D chromatin conformation and gene regulation during <i>Drosophila</i> dorsoventral patterning" has now been seen by 3 referees. You will see from their comments below that while they find your work of interest, some important points are raised. We are interested in the possibility of publishing your study in Nature Genetics, but would like to consider your response to these concerns in the form of a revised manuscript before we make a final decision on publication.

Reviewer #1 is positive overall. Their main concern is that the cell populations analyzed are not pure populations (i.e., the mutants mainly enrich for a specific tissue type). The reviewer thinks that single-cell Hi-C analysis or additional modeling could be used to address this point.
Reviewer #2 thinks this is a strong analysis and seems supportive of publication. Their comments can be addressed textually.
In contrast, reviewer #3 feels that conceptually the results are very similar to what has been reported before: that 3D conformation doesn't vary substantially across tissues. This is a salient criticism, but I think that the reviewer might be potentially disregarding two aspects here: data resolution (looking also at loops, instead of just TADs) and in vivo tissues versus cultured cells. Reviewer #3 doesn't have many constructive suggestions, unfortunately.

We invite you to revise your manuscript taking into account all reviewer comments. Please highlight

all changes in the manuscript text file. At this stage we will need you to upload a copy of the manuscript in MS Word .docx or similar editable format.

We are committed to providing a fair and constructive peer-review process. Do not hesitate to contact me if there are specific requests from the reviewers that you believe are technically impossible or unlikely to yield a meaningful outcome.

When revising your manuscript:

*1) Include a “Response to referees” document detailing, point-by-point, how you addressed each referee comment. If no action was taken to address a point, you must provide a compelling argument. This response will be sent back to the referees along with the revised manuscript.

*2) If you have not done so already please begin to revise your manuscript so that it conforms to our Article format instructions, available
here.
Refer also to any guidelines provided in this letter.

*3) Include a revised version of any required Reporting Summary:
https://www.nature.com/documents/nr-reporting-summary.pdf
It will be available to referees (and, potentially, statisticians) to aid in their evaluation if the manuscript goes back for peer review.
A revised checklist is essential for re-review of the paper.

Please be aware of our guidelines on digital image standards.

Please use the link below to submit your revised manuscript and related files:

[REDACTED]

Note: This URL links to your confidential home page and associated information about manuscripts you may have submitted, or that you are reviewing for us. If you wish to forward this email to co-authors, please delete the link to your homepage.

Please do not hesitate to contact me if you have any questions or would like to discuss these revisions further.

Nature Genetics is committed to improving transparency in authorship. As part of our efforts in this direction, we are now requesting that all authors identified as ‘corresponding author’ on published papers create and link their Open Researcher and Contributor Identifier (ORCID) with their account on the Manuscript Tracking System (MTS), prior to acceptance. ORCID helps the scientific community achieve unambiguous attribution of all scholarly contributions. You can create and link your ORCID from the home page of the MTS by clicking on ‘Modify my Springer Nature account’. For more information please visit please visit <a
href="http://www.springernature.com/orcid">www.springernature.com/orcid.

We look forward to seeing the revised manuscript and thank you for the opportunity to review your

work.

Sincerely,

Tiago

Tiago Faial, PhD
Senior Editor
Nature Genetics
https://orcid.org/0000-0003-0864-1200

Reviewers' Comments:

Reviewer #1:
Remarks to the Author:
Ing-Simmons and colleagues examined genome architecture in the developing Drosophila embryo, using Toll pathway mutations to generate embryos enriched for mesoderm, neurectoderm, and dorsal ectoderm. They determine that while transcription profiles and chromatin state change significantly between these three mutants, chromatin architecture does not, leading them to conclude that tissue-specific chromatin architecture is not required for tissue-specific gene expression.

It seems clear from these data that gene expression and chromatin state are more sensitive to population-level changes in the ratios of different cell types than is genome architecture. This is an important conclusion.

My only major concern is that the model system is, as the authors acknowledge, somewhat messy because these mutants represent enrichments/depletions rather than pure populations. In the light of this limitations, the conclusions may be overly strong. The key question is whether this is because genome architecture is more similar/consistent across cell types or because it is more variable within any given cell type? Ideally the authors would address this by doing a single-cell Hi-C experiment, but it may be possible to instead use modelling on the basis of the Hi-C data.

Minor comments:
1) Figure 3 shows some really odd saddle plots that seem to have internal structure usually averaged out in saddle plots. Is this a representative frame instead? If not, what is generating the internal structure?
2) In figure 4, using one of the chromosomes with structural rearrangements (i.e. 3R as in Figure S4B) would be a more persuasive way to prove the point than using chrX which does not have any structural rearrangements.
3) In the discussion of the segments which show architectural differences without structural rearrangements (Figure S5), it appears that these regions are also bereft of differentially expressed genes or enhancer marks. Clarifying this in the text would reduce confusion, since if there were a

handful of cases where tissue-specific genome architecture correlated with tissue-specific expression, these might be very interesting examples to study.
4) In figure S7, is the color bar on a log scale? If so, please specify.

Reviewer #2:
Remarks to the Author:
Understanding the relationship between chromatin conformation and expression is important and unresolved. There are clearly a few examples of genes that take advantage of the 3D (or 4D) structure for regulatory interactions between distant sites in a 2D linear distance along a chromosome, but it is much less clear how important this is more generally. The combination of a well-established available and new datasets provides a nice window to the problem. The largely negative results are more important than is usually the case, given how widely accepted the dogma on how important these distant contacts are for gene regulation -- it seems at least as likely that these conserved 3D structures are primarily for packing DNA. This is a strong analysis.

My only criticism is the presentation, but these are really discretionary. I'm trying to be helpful rather than create a lot of work. The authors write very well, but densely. They are showing a lot of data and analysis without explaining it well enough to keep me from getting lost without requiring moving around a lot between the results and the methods, as well as the main text and the supplement. This paper took me about twice as long as I scheduled. This is not the only reason my review is late (apologies), but it did contribute. This would be a hard slog for a graduate student journal club. Making a paper longer is almost never a good idea, but I think that making this one longer (or with a significant supplement if the authors like the flow) would allow the authors to make it clearer. This should focus on the beginning of the results. Let the reader dip in a toe before plunging in. The authors will have a more impactful paper, if they make it more accessible.

Science

1) The authors are clear about the reuse of other datasets, which is laudable. Some discussion of how well those datasets would be expected to align would be useful. This might not be the type of watermarking one would use in a fully designed set of experiments, but the reader should have more information about dataset comparability. Not making a statement will cause readers to wonder about comparing 2-4 hpf ChIP with 3-4 hpf Hi-C. What is the replication level in all the datasets? A simple table of datasets used in this study would help, but given how fast development proceeds, a sentence of two up front in the main text would also be useful.

2) It would be a good idea to submit the code to zenodo or some other such repository.

3) Consider including two papers that support the argument that gross architecture does not generically contribute to gene expression PMID: 21151342 and 27599372.

4) It seems to me that the conclusion that flows most directly from the data is that the transcriptional activity of the embryo has no effect on the spatial organization of the genome. I'd emphasize this positive result more.

Presentation

5) While the concept of finding target enhancers for the analysis (line 125) is logical, I had to draw out a flow chart to get an idea of exactly how this was done. Some type of visual like that might help other readers.

6) Define units like CPM and TPM at first occurrence in figures rather than at the end in the methods.

7) The synthesis outlined in Figure 1 F and G are potentially nice examples at the domain-level, but it is hard to line-up the genes with the tracks, the shaded regions are hard to distinguish, and the * appears to be above, below, or to the right or left of the gene being identified. Really hard to tell. Maybe the genes of interest could be blown up a bit?

8) Figure 1 is lacking a global view of the complex selection process. What do relationships among these data-types look like globally? I'd include the supplemental upset plot in Figure 1.

9) For the new scRNA-seq data, while the quality of the data look outstanding, there is scant detail in the manuscript. The serious reader will want to know if the cell-type calls are robust. It would be useful to callout a few clear examples in the main text, rather than sending the reader to a supplemental table. Personally, I would prefer some sort of a differential expression heatmap to visualize the differences among the cell types.

10) The mesoderm clusters in particular need of some validation in the main text. This should include specific genes that contribute to the depletion of the mesoderm clusters in the mutants (Fig. 2B), or the striking pattern of enhancers (Fig. 2C). This should extend beyond the few examples given later (doc, net) and include some of the more famous genes that the reader will be familiar with.

11) Some details on the methods would not take much space. For example, when calling a cell type, is that positive only? Present and excluded?

Reviewer #3:
Remarks to the Author:
The main message of this study, that "cell type-specific gene regulation does not require cell type-specific chromatin conformation" is not new, and the approach does not provide further detail than previous studies. In other words, the resolution used in this study is not significantly higher than used in previous studies that have also shown that different cell types, with cell-specific transcriptional programs, have similar chomatin organization at the level of topological domains (e.g. Nora et al 2012, one of the first papers reporting TADs, in which the authors showed similar topological organization for embryonic stem cells, fibroblasts and neural precursors; or Smith, Lajoie, Jain and Dekker 2016, in which the authors analysed the topological organization of the CFTR locus across five different cell lines of different tissue origins; and other many examples, including Bonev et al 2017 and other studies cited in this manuscript).

It would strengthen the manuscript if the authors explained why the need to "identify candidate tissue-specific regulatory elements involved in dorsoventral patterning"

I think the authors should be more carefully with wording:

- In the Abstract: "Here, using the Drosophila melanogaster dorsoventral patterning system as a model, we demonstrate the independence of 3D chromatin organisation and developmental gene regulation." I don't think that we can infer the independence of 3D chromatin organisation and developmental gene regulation, in such global terms, based only on the model of dorsoventral patterning.

- The authors use the expression "3D chromatin organization" to refer to _________; this is influenced by the technique used. Other methods probing chromatin organization have shown that different types of organization can be revealed by different methods – in this study, only those types revealed by Hi-C are being analysed, so the question remains open for the other types of chromatin organization.

- "we observed that dorsoventral patterning genes and their regulatory elements are located within the same self-interacting domains (Fig. 1F, grey shaded region; Fig. 1G)." Figures 1F and 1G show two genomic regions – do these include all dorsoventral patterning gens and their regulatory elements, as the sentence suggests?

- "our results demonstrate that overall genome organisation at the level of compartments, domains, and chromatin loops is similar across tissues in cellular blastoderm embryos." The results demonstrate that overall genome organization is similar across *genotypes*, not across tissues, as tissues are not probed separately in this study.

- "we found no evidence for widespread enrichment of interactions between enhancers and their target promoters. This is in line with "transcriptional hub" models, in which transient or indirect contacts with a regulatory element are sufficient to activate transcription" I think this reasoning is misleading and not well-founded; no one has yet shown how transcriptional hubs/microenvironments/etc relate to topological organization – do those transient interactions leave really "no trace" in 3C-based techniques? It is still unclear. Moreover, one of the first models of "transcriptional hubs", the active chromatin hub (see e.g. de Laat and Grosveld 2003) was actually based on the identification of interactions between cis-regulatory elements.

Author Rebuttal to Initial comments

We would like to thank all the reviewers for the constructive feedback and helpful suggestions. Following these, we have revised the text and figures for clarity, particularly regarding the methodology and how the datasets used relate to each other.

In addition, in this revised version we have included additional datasets that further strengthen the conclusions of this work. Firstly, we have included additional sequencing for the original libraries, increasing the resolution of these datasets, and performed insulation score analysis around domain boundaries and regulatory elements to further confirm the maintenance of chromatin conformation at these regions (new Figure S7). Secondly, we have included newly generated Micro-C data for wild type embryos and *gd*^{7} mutant embryos which provides an increased resolution of up to 500 bp (Figure 5). To our knowledge this is the first available Micro-C data for *Drosophila* and reveals a striking level detail in the chromatin conformation of these embryos. Importantly, our results are strongly supported by this higher-resolution analysis, indicating that the Hi-C results are not limited by resolution. Thirdly, we also have included Hi-C data for later stage embryos (Figure S9) that demonstrates that our results are not solely due to the early developmental stage considered in the initial submission of the work.

To address the important question of whether cell type heterogeneity in these samples causes us to not be able to detect tissue-specific chromatin conformation, we have also carried out analysis of synthetic mixtures of Hi-C data from different samples. These support our conclusions that the chromatin conformation is conserved across cell types. Finally, we wish to note that we made minor modifications to the parameters for candidate enhancer identification, which lead to increased numbers of candidate tissue-specific enhancers, in particular for *Toll*$^{rm9/rm10}$; therefore, the numbers of enhancers in some figures have changed, although the overall conclusions are unaffected.

We have responded to each reviewer comment individually below. We have included reviewer comments in blue italic text with our responses in black text, and excerpts from the revised manuscript in black bold text.

Reviewer #1:

Remarks to the Author:

Ing-Simmons and colleagues examined genome architecture in the developing Drosophila embryo, using Toll pathway mutations to generate embryos enriched for mesoderm, neurectoderm, and dorsal ectoderm. They determine that while transcription profiles and chromatin state change significantly between these three mutants, chromatin architecture does not, leading them to conclude that tissue-specific chromatin architecture is not required for tissue-specific gene expression.

It seems clear from these data that gene expression and chromatin state are more sensitive to population-level changes in the ratios of different cell types than is genome architecture. This is an important conclusion.

My only major concern is that the model system is, as the authors acknowledge, somewhat messy because these mutants represent enrichments/depletions rather than pure populations. In the light of this limitations, the conclusions may be overly strong. The key question is whether this is because genome architecture is more similar/consistent across cell types or because it is more variable within any given cell type? Ideally the authors would address this by doing a single-cell Hi-C experiment, but it may be possible to instead use modelling on the basis of the Hi-C data.

We thank the reviewer for recognising the importance of the conclusions that can be drawn from this data and for the helpful suggestions.

We agree that the question of whether heterogeneity in cell identities within mutant embryos masks tissue-specific changes in chromatin organisation is an important one. We first wish to clarify that we acknowledge that genome architecture varies from cell to cell within a population. We have clarified in the text (lines 377-381) that we don't wish to claim that chromatin conformation is identical in all cells across the embryo. Rather, we believe that cells from different tissues within the embryo have chromatin conformations drawn from the same population of possible conformations, rather than different tissues having different ensembles of possible conformations. This section reads as follows:

"Hi-C provides information about the average conformation across a population of hundreds of thousands of nuclei, which contain dynamic ensembles of different 3D conformations [89,91–99]. While our scRNA-seq results indicate that the mutant embryos contain a range of different cell types, we believe that our results indicate that the 3D chromatin structures in these cell types are drawn from the same population of possible conformations."

We agree that single-cell resolution chromatin conformation data would be ideal to address this question. Current single-cell Hi-C technologies are unfortunately extremely sparse and therefore do not allow assessment of enhancer-promoter interactions at the single-cell level, and only provide limited information about domain organisation in single cells. Therefore, we do not believe that single-cell Hi-C would be an appropriate approach to address this concern. Regarding the suggestion to perform modelling using the Hi-C data, we are not sure that 3D modelling would be useful here due to the inherent difficulty of obtaining robust ensemble 3D models from average bulk Hi-C data.

However, imaging approaches can provide information about chromatin conformation in single cells at high genomic resolution. A co-submitted manuscript from the Lagha and Nollmann labs (preprint: Espinola, Götz et al., *biorXiv*, 2020, doi: 10.1101/2020.07.07.191015) uses Hi-M, a high-resolution imaging approach, to also address the question of whether chromatin conformation at developmentally regulated genes varies across tissues in the Drosophila embryo around ZGA. Strikingly, they find that regulatory interactions are established across tissues, independent of cell fate, in agreement with our findings. Therefore, we believe that our results accurately reflect the similarity of chromatin conformation across different tissues within the embryo and that tissue-specific structures are not masked or diluted by heterogeneity in cell identities within the mutant embryos. We discuss these findings in more detail in the revised discussion (lines 381-388): **"This is supported by results from a recent preprint [100] which analyses the structure of the *Doc* and *sna* loci in *Drosophila* embryos using Hi-M, a high-resolution single-cell imaging approach. Strikingly, this orthogonal technique also reveals chromatin organisation that is consistent across different tissues in the embryo, despite differential expression of these genes. However, Hi-M finds enriched enhancer-enhancer and enhancer-promoter interactions at the *sna* locus which are not apparent in Hi-C or Micro-C data (Fig. S6D, S7D). This discrepancy will require further study to better understand the biological basis of these differences and the strengths and limitations of each approach."**

Minor comments:
1) Figure 3 shows some really odd saddle plots that seem to have internal structure usually averaged out in saddle plots. Is this a representative frame instead? If not, what is generating the internal structure?

The "+" structure in these saddle plots is due to a relatively large fraction of the genome having an eigenvector value of zero; this fraction of the genome then occupies multiple rows/columns in the aggregate matrix which all have the same average interaction tendencies. We agree that this difference from typical saddle plots is confusing. To address this, we have implemented two improvements to the visualisation to remove this artefact: first, we masked pericentromeric heterochromatin marked by H3K9me3 in the Hi-C matrices before carrying out compartment aggregate analysis. Second, we collapsed all rows/columns with identical eigenvector values into a single row/column in the aggregate matrix. The resulting plots (Figures 3 and S7) better reflect the meaningful genomic interactions that contribute to compartmentalisation and are more similar to typical mammalian saddle plots.

2) In figure 4, using one of the chromosomes with structural rearrangements (i.e. 3R as in Figure S4B) would be a more persuasive way to prove the point than using chrX which does not have any structural rearrangements.

Thank you for the suggestion. The revised version of Figure 4 now shows chr 3R, highlighting a region which has rearrangements on balancer chromosomes present in Tollrm9/10 and Toll10B embryos.

3) In the discussion of the segments which show architectural differences without structural rearrangements (Figure S5), it appears that these regions are also bereft of differentially expressed genes or enhancer marks. Clarifying this in the text would reduce confusion, since if there were a

handful of cases where tissue-specific genome architecture correlated with tissue-specific expression, these might be very interesting examples to study.

We have clarified this in the text (lines 227-230): **"Of two example loci on chromosome 2 with changes in chromatin organisation identified by CHESS in *Toll[10B]* embryos, while one is close to a cluster of *Toll[10B]*-specific enhancers (Fig. S5B), the other occurs in a region devoid of H3K27ac, H3K27me3 or RNA-seq signal in any of the mutant genotypes (Fig. S5D)."**

4) In figure S7, is the color bar on a log scale? If so, please specify.

Yes, the colour bar label should have read "log2(observed/expected contact probability)". We have corrected this in the revised figure (now Fig 5 D/E).

Reviewer #2:

Remarks to the Author:

Understanding the relationship between chromatin conformation and expression is important and unresolved. There are clearly a few examples of genes that take advantage of the 3D (or 4D) structure for regulatory interactions between distant sites in a 2D linear distance along a chromosome, but it is much less clear how important this is more generally. The combination of a well-established available and new datasets provides a nice window to the problem. The largely negative results are more important than is usually the case, given how widely accepted the dogma on how important these distant contacts are for gene regulation -- it seems at least as likely that these conserved 3D structures are primarily for packing DNA. This is a strong analysis.

My only criticism is the presentation, but these are really discretionary. I'm trying to be helpful rather than create a lot of work. The authors write very well, but densely. They are showing a lot of data and analysis without explaining it well enough to keep me from getting lost without requiring moving around a lot between the results and the methods, as well as the main text and the supplement. This paper took me about twice as long as I scheduled. This is not the only reason my review is late (apologies), but it did contribute. This would be a hard slog for a graduate student journal club. Making a paper longer is almost never a good idea, but I think that making this one longer (or with a significant supplement if the authors like the flow) would allow the authors to make it clearer. This should focus on the beginning of the results. Let the reader dip in a toe before plunging in. The authors will have a more impactful paper, if they make it more accessible.

Thank you for the positive review and for the helpful comments. We have sought additional feedback on the manuscript density and clarity and revised the text accordingly; we hope that the revised text is clearer and more accessible.

Science

1) The authors are clear about the reuse of other datasets, which is laudable. Some discussion of how well those datasets would be expected to align would be useful. This might not be the type of watermarking one would use in a fully designed set of experiments, but the reader should have more information about dataset comparability. Not making a statement will cause readers to wonder about comparing 2-4 hpf ChIP with 3-4 hpf Hi-C. What is the replication level in all the datasets? A simple table of datasets used in this study would help, but given how fast development proceeds, a sentence of two up front in the main text would also be useful.

We thank the reviewer for the suggestion. As suggested, we have included a table specifying the datasets used and the number of replicates (Table S4). We have also included a new Fig. 1A with a schematic of development depicting how the timing of the datasets relates to each other., and described these relationships in more detail in the results section.

2) It would be a good idea to submit the code to zenodo or some other such repository.

We have added the analysis code to a public github respository (https://github.com/vaquerizaslab/Ing-Simmons_et_al_dorsoventral_3D_genome/) and archived it with Zenodo (DOI: 10.5281/zenodo.4272002).

3) Consider including two papers that support the argument that gross architecture does not generically contribute to gene expression PMID: 21151342 and 27599372.

We have included references to these papers in the introduction (line 52).

4) It seems to me that the conclusion that flows most directly from the data is that the transcriptional activity of the embryo has no effect on the spatial organization of the genome. I'd emphasize this positive result more.

Thank you for the suggestion. As suggested, we have revised the discussion to emphasise this point more (lines 350-352): **"Our results do not support this model, as we find no evidence that new domains are driven by changes in chromatin state or by active expression of developmentally-regulated genes. In contrast this supports the idea that, similarly to mammalian domain architecture, additional factors such as insulator proteins modulate domain organisation in *Drosophila* [2,89]. Therefore, we do not believe that active transcription is the key determinant of 3D chromatin organisation in this system."**

Presentation

5) While the concept of finding target enhancers for the analysis (line 125) is logical, I had to draw out a flow chart to get an idea of exactly how this was done. Some type of visual like that might help other readers.

We have added a schematic depiction of enhancer assignment to Figure S1 (new panel F) and hope that this illustration helps to explain the process.

6) Define units like CPM and TPM at first occurrence in figures rather than at the end in the methods.

We have ensured that units are defined in the figure legends where they first occur.

*7) The synthesis outlined in Figure 1 F and G are potentially nice examples at the domain-level, but it is hard to line-up the genes with the tracks, the shaded regions are hard to distinguish, and the * appears to be above, below, or to the right or left of the gene being identified. Really hard to tell. Maybe the genes of interest could be blown up a bit?*

We have revised these visualisations and hope that they are now clearer. We have highlighted the genes of interest by changing the colour of the gene(s) of interest to black and including the gene name in the figure (in all applicable figures) rather than indicating them with asterisks. To improve clarity of the shaded regions in Fig 1G (previously Fig 1F) we have used more intense colours for the shading and additionally outlined the shaded areas.

8) Figure 1 is lacking a global view of the complex selection process. What do relationships among these data-types look like globally? I'd include the supplemental upset plot in Figure 1.

We have included a new Fig 1A with a schematic representation of Drosophila embryonic development to illustrate how the different datasets were collected with respect to developmental timing and how these datasets relate each other. This is also discussed in the revised text (lines 111-114: **"Embryos collected at 2-4 hpf will largely consist of embryos in the late cellular blastoderm stage and undergoing gastrulation, thus targeting our timepoint of interest. Note that while H3K27ac is established from nc12 onwards, H3K27me3 is only present from mid nc14 [65]"** .

We have also clarified the process by which tissue-specific enhancers are selected in the revised Fig 1B (now Fig 1C). We hope that this makes the relationship between datasets and the enhancer selection process clearer.

We are concerned that including the UpSet plot in Fig.1 will significantly increase the complexity of the figure and the message, and have therefore decided to keep it in Fig S2. The key conclusion from Fig. S2 is that our findings are robust to different choices of candidate enhancers, as we mention in lines 141-142. For the main figures, we use our enhancer sets as this allows examination of *Toll*$^{rm9/rm10}$ in addition to *gd*^{7} and *Toll*^{10B}.

9) For the new scRNA-seq data, while the quality of the data look outstanding, there is scant detail in the manuscript. The serious reader will want to know if the cell-type calls are robust. It would be useful to callout a few clear examples in the main text, rather than sending the reader to a supplemental table. Personally, I would prefer some sort of a differential expression heatmap to visualize the differences among the cell types.

Thank you for the suggestion; we agree that a differential expression heatmap is a useful way to visualise expression patterns across clusters and have included a heatmap showing expression of the top ten marker genes per cluster in the revised Figure 2 (new panel B). We have also indicated selected marker genes in the heatmap and referred to these in the text (lines 158-162): **"We identified clusters representing mesoderm (*twi*, *Mlc2*, *Mdr49*), ectoderm (*ab*, *sca*, *SoxN*), in addition to other cell populations such as amnioserosa (*Ance*, *peb*), terminal regions of the embryo (*fkh*), pole cells (*pgc*), haemocytes (*PPO1*), and trachea precursors (Osiris gene cluster)."**

10) The mesoderm clusters in particular need of some validation in the main text. This should include specific genes that contribute to the depletion of the mesoderm clusters in the mutants (Fig. 2B), or the striking pattern of enhancers (Fig. 2C). This should extend beyond the few examples given later (doc, net) and include some of the more famous genes that the reader will be familiar with.

We have indicated selected marker genes, including several mesoderm marker genes e.g. *twi*, in the new heatmap of differential expression across clusters (Fig 2B). We have additionally mentioned these selected top marker genes in the text (lines 158-162).

11) Some details on the methods would not take much space. For example, when calling a cell type, is that positive only? Present and excluded?

We are not completely sure what the reviewer is referring to here. With regards to cluster identification, we have included additional detail in the results section, specifically that marker genes are upregulated in the given clusters (line 157).

Reviewer #3:

Remarks to the Author:

The main message of this study, that "cell type-specific gene regulation does not require cell type-specific chromatin conformation" is not new, and the approach does not provide further detail than previous studies. In other words, the resolution used in this study is not significantly higher than used in previous studies that have also shown that different cell types, with cell-specific transcriptional programs, have similar chromatin organization at the level of topological domains (e.g. Nora et al 2012, one of the first papers reporting TADs, in which the authors showed similar topological organization for embryonic stem cells, fibroblasts and neural precursors; or Smith, Lajoie, Jain and Dekker 2016, in which the authors analysed the topological organization of the CFTR locus across five different cell lines of different tissue origins; and other many examples, including Bonev et al 2017 and other studies cited in this manuscript).

Thank you for your constructive feedback.

While it is true that many studies have concluded that 3D chromatin organisation is similar across different cell types using *in vitro* approaches, other studies have identified cell type specific chromatin organisation at the level of domains, loops, or enhancer-promoter interactions (including Bonev et al., 2017; Chathoth and Zabet, 2019; Joshi et al., 2015; Kragesteen et al., 2018; Le Dily et al., 2014; Oudelaar et al., 2020; Schmitt et al., 2016). Therefore, the question still stands as to whether 3D chromatin conformation is necessary for tissue-specific expression in an *in vivo* system. We believe

that our study is the first to investigate the relationship between cell type-specific gene regulation and chromatin conformation genome-wide in different tissues within a developing embryo, rather than comparing cell lines or differentiated tissues. Specifically, here we use a genetic system that perturbs embryonic development, to obtain cell populations that can be used to represent different tissues. In addition, in comparison to earlier studies in *Drosophila* embryos (e.g. Ghavi-Helm et al. 2014, which used 4C-seq on a limited set of genomic loci), by using Hi-C we are able to assess chromatin conformation features across the whole genome and at multiple levels, including both domains and enhancer-promoter interactions.

In addition, as described above, this revised version of the manuscript includes analysis of Micro-C data that provides a strikingly increased level of resolution and that we believe to be the first Micro-C available in *Drosophila*. Our manuscript also includes scRNA-seq data for a classic developmental model system that, for the first time characterises the level of heterogeneity of this system and will be an extremely valuable resource for the community.

It would strengthen the manuscript if the authors explained why the need to "identify candidate tissue-specific regulatory elements involved in dorsoventral patterning"

We have added an additional explanatory sentence to the start of the results section to explain that identification of candidate regulatory elements is a key first step in examining the 3D chromatin context of dorsoventral gene regulation genome-wide.

I think the authors should be more carefully with wording:

- In the Abstract: "Here, using the Drosophila melanogaster dorsoventral patterning system as a model, we demonstrate the independence of 3D chromatin organisation and developmental gene regulation." I don't think that we can infer the independence of 3D chromatin organisation and developmental gene regulation, in such global terms, based only on the model of dorsoventral patterning.

We have revised this sentence to clarify that our results apply at this developmental stage.

- The authors use the expression "3D chromatin organization" to refer to _________; this is influenced by the technique used. Other methods probing chromatin organization have shown that different types of organization can be revealed by different methods – in this study, only those types revealed by Hi-C are being analysed, so the question remains open for the other types of chromatin organization.

We are not quite sure what the reviewer means here. We agree that different methods of investigating chromatin conformation have different strengths. We would like to bring the reviewer's attention to a co-submitted manuscript from the Lagha and Nollmann labs (preprint: Espinola, Götz et al., *biorXiv*, 2020, doi: 10.1101/2020.07.07.191015) which uses a high-resolution imaging approach to investigate regulatory interactions in single cells in wild type Drosophila embryos. Strikingly, this orthogonal approach also reveals conserved chromatin conformation of dorsoventral patterning genes across tissues regardless of gene expression or cell fate. We discuss these results in more detail in the revised discussion (lines 381-388): **"This is supported by results from a recent preprint [100] which analyses the structure of the *Doc* and *sna* loci in *Drosophila* embryos using Hi-M, a high-resolution single-cell imaging approach. Strikingly, this orthogonal technique also reveals chromatin organisation that is consistent across different tissues in the embryo, despite differential expression of these genes. However, Hi-M finds enriched enhancer-enhancer and enhancer-promoter interactions at the *sna* locus which are not apparent in Hi-C or Micro-C data (Fig. S6D, S7D). This discrepancy will require further study to better understand the biological basis of these differences and the strengths and limitations of each approach."**

- "we observed that dorsoventral patterning genes and their regulatory elements are located within the same self-interacting domains (Fig. 1F, grey shaded region; Fig. 1G)." Figures 1F and 1G show two genomic regions – do these include all dorsoventral patterning gens and their regulatory elements, as the sentence suggests?

We have revised this sentence for clarity: **"we observed that dorsoventral patterning genes are located within self-interacting domains, along with their assigned regulatory elements (e.g. Fig. 1G, grey shaded region; Fig. 1H)."** (lines 133-135).

*- "our results demonstrate that overall genome organisation at the level of compartments, domains, and chromatin loops is similar across tissues in cellular blastoderm embryos." The results demonstrate that overall genome organization is similar across *genotypes*, not across tissues, as tissues are not probed separately in this study.*

We have revised this sentence for clarity; it now reads **"our results demonstrate that overall genome organisation at the level of compartments, domains, and chromatin loops is highly similar across genotypes, suggesting that it is conserved across tissues in cellular blastoderm embryos"** (lines 205-206).

- "we found no evidence for widespread enrichment of interactions between enhancers and their target promoters. This is in line with "transcriptional hub" models, in which transient or indirect contacts with a regulatory element are sufficient to activate transcription" I think this reasoning is misleading and not well-founded; no one has yet shown how transcriptional hubs/microenvironments/etc relate to topological organization – do those transient interactions leave really "no trace" in 3C-based techniques? It is still unclear. Moreover, one of the first models of "transcriptional hubs", the active chromatin hub (see e.g. de Laat and Grosveld 2003) was actually based on the identification of interactions between cis-regulatory elements.

We agree that the use of the term "transcriptional hub" could be confusing, as this term has been used in different ways in the literature over time. Therefore, we have removed this terminology.

We agree that it is unclear whether transient enhancer-promoter interactions (e.g. within a nuclear microenvironment / transcriptional hub) will be detectable in 3C techniques; indeed, this is one of the questions we sought to address with this work. Our results indicate that there is no substantial enrichment of enhancer-promoter interactions as detected by Hi-C or Micro-C in the tissues where the enhancers are active. Importantly, this conclusion is supported by independent work using imaging approaches (Espinola, Götz et al., *biorXiv*, 2020, doi: 10.1101/2020.07.07.191015), as described above and in the revised discussion (lines 381-388).

natureresearch

Decision Letter, first revision:

Our ref: NG-A55249R

4th Dec 2020

Dear Juanma,

Thank you for submitting your revised manuscript "Independence of 3D chromatin conformation and gene regulation during <i>Drosophila</i> dorsoventral patterning" (NG-A55249R). It has now been seen by the original referees and their comments are below. The reviewers find that the paper has improved in revision, and therefore we will be happy in principle to publish it in Nature Genetics, pending minor revisions to comply with our editorial and formatting guidelines.

** Note that I will send you a checklist detailing these editorial and formatting requirements in about a week. Please do not finalize your revisions or upload the final materials until you receive this additional information.**

In recognition of the time and expertise our reviewers provide to Nature Genetics's editorial process, we would like to formally acknowledge their contribution to the external peer review of your manuscript entitled "Independence of 3D chromatin conformation and gene regulation during <i>Drosophila</i> dorsoventral patterning". For those reviewers who give their assent, we will be publishing their names alongside the published article.

While we prepare these instructions, we encourage the Corresponding Author to begin to review and collect the following:

-- Confirmation from all authors that the manuscript correctly states their names, institutional affiliations, funding IDs, consortium membership and roles, author or collaborator status, and author contributions.

-- Declarations of any financial and non-financial competing interests from any author. For the sake of transparency and to help readers form their own judgment of potential bias, the Nature Research Journals require authors to declare any financial and non-financial competing interests in relation to the work described in the submitted manuscript. This declaration must be complete, including author initials, in the final manuscript text.

If you have any questions as you begin to prepare your submission please feel free to contact our Editorial offices at genetics@us.nature.com. We are happy to assist you.

Thank you again for your interest in Nature Genetics.

Sincerely,

Tiago

natureresearch

Tiago Faial, PhD
Senior Editor
Nature Genetics
https://orcid.org/0000-0003-0864-1200

Reviewer #1 (Remarks to the Author):

The authors have addressed our points quite comprehensively. No further questions.

Reviewer #2 (Remarks to the Author):

Thank you for an interesting paper and taking the effort to respond to the comments.

Reviewer #3 (Remarks to the Author):

The revised manuscript has satisfied my original issues. Previously, my main concern was the resolution of their enhancer-promoter assays and the subsequent conclusions. The inclusion of Micro-C, which is consistent with the rest of their findings, has resolved this issue.

ORCID

Nature Genetics is committed to improving transparency in authorship. As part of our efforts in this direction, we are now requesting that all authors identified as ‘corresponding author’ create and link their Open Researcher and Contributor Identifier (ORCID) with their account on the Manuscript Tracking System (MTS) prior to acceptance. ORCID helps the scientific community achieve unambiguous attribution of all scholarly contributions. For more information please visit http://www.springernature.com/orcid

For all corresponding authors listed on the manuscript, please follow the instructions in the link below to link your ORCID to your account on our MTS before submitting the final version of the manuscript. If you do not yet have an ORCID you will be able to create one in minutes.
https://www.springernature.com/gp/researchers/orcid/orcid-for-nature-research

IMPORTANT: All authors identified as ‘corresponding author’ on the manuscript must follow these instructions. Non-corresponding authors do not have to link their ORCIDs but are encouraged to do so. Please note that it will not be possible to add/modify ORCIDs at proof. Thus, if they wish to have their ORCID added to the paper they must also follow the above procedure prior to acceptance.

To support ORCID's aims, we only allow a single ORCID identifier to be attached to one account. If you have any issues attaching an ORCID identifier to your MTS account, please contact the Platform Support Helpdesk.

Final Decision Letter:

21st Jan 2021

Dear Juanma,

I am delighted to say that your manuscript "Independence of chromatin conformation and gene regulation during <i>Drosophila</i> dorsoventral patterning" has been accepted for publication in an upcoming issue of Nature Genetics.

Prior to setting your manuscript, we may make minor changes to enhance the lucidity of the text and with reference to our house style. We therefore ask that you examine the proofs most carefully to ensure that we have not inadvertently altered the sense of your text in any way.

Once your manuscript is typeset and you have completed the appropriate grant of rights, you will receive a link to your electronic proof via email with a request to make any corrections within 48 hours. If, when you receive your proof, you cannot meet this deadline, please inform us at rjsproduction@springernature.com immediately.

Your paper will be published online after we receive your corrections and will appear in print in the next available issue. You can find out your date of online publication by contacting the Nature Press Office (press@nature.com) after sending your e-proof corrections. Now is the time to inform your Public Relations or Press Office about your paper, as they might be interested in promoting its publication. This will allow them time to prepare an accurate and satisfactory press release. Include your manuscript tracking number (NG-A55249R1) and the name of the journal, which they will need when they contact our Press Office.

Before your paper is published online, we shall be distributing a press release to news organizations worldwide, which may very well include details of your work. We are happy for your institution or funding agency to prepare its own press release, but it must mention the embargo date and Nature Genetics. Our Press Office may contact you closer to the time of publication, but if you or your Press Office have any enquiries in the meantime, please contact press@nature.com.

Acceptance is conditional on the data in the manuscript not being published elsewhere, or announced in the print or electronic media, until the embargo/publication date. These restrictions are not intended to deter you from presenting your data at academic meetings and conferences, but any enquiries from the media about papers not yet scheduled for publication should be referred to us.

Nature Genetics is a transformative open access journal. In approximately 10 business days you will receive an email with a link to choose the appropriate publishing options for your paper and our Author Services team will be in touch regarding any additional information that may be required.

Please note that Nature Research offers an immediate open access option only for papers that were first submitted after 1 January, 2021.

You will not receive your proofs until the publishing agreement has been received through our system.

If you have any questions about our publishing options, costs, Open Access requirements, or our legal forms, please contact ASJournals@springernature.com

In the event that you choose to publish under the subscription model, Nature Research allows authors to self-archive the accepted manuscript (the version post-peer review, but prior to copy-editing and typesetting) on their own personal website and/or in an institutional or funder repository where it can be made publicly accessible 6 months after first publication, in accordance with our self-archiving policy. Please review our self-archving policy for more information.

Several funders require deposition the accepted manuscript (AM) to PubMed Central or Europe PubMed Central. To enable compliance with these requirements, Nature Research therefore offers a free manuscript deposition service for original research papers supported by a number of PMC/EPMC participating funders. If you do not choose to publish immediate open access, we can deposit the accepted manuscript in PMC/Europe PMC on your behalf, if you authorise us to do so.

If you have posted a preprint on any preprint server, please ensure that the preprint details are updated with a publication reference, including the DOI and a URL to the published version of the article on the journal website.

To assist our authors in disseminating their research to the broader community, our SharedIt initiative provides you with a unique shareable link that will allow anyone (with or without a subscription) to read the published article. Recipients of the link with a subscription will also be able to download and print the PDF.

As soon as your article is published, you will receive an automated email with your shareable link.

You can now use a single sign-on for all your accounts, view the status of all your manuscript submissions and reviews, access usage statistics for your published articles and download a record of your refereeing activity for the Nature journals.

An online order form for reprints of your paper is available at https://www.nature.com/reprints/author-reprints.html. Please let your coauthors and your institutions' public affairs office know that they are also welcome to order reprints by this method.

Sincerely,

natureresearch

Tiago

Tiago Faial, PhD
Senior Editor
Nature Genetics
https://orcid.org/0000-0003-0864-1200

Click here if you would like to recommend Nature Genetics to your librarian
http://www.nature.com/subscriptions/recommend.html#forms

** Visit the Springer Nature Editorial and Publishing website at www.springernature.com/editorial-and-publishing-jobs for more information about our career opportunities. If you have any questions please click here.**
